# TRIP6 antagonizes the recruitment of A20 and CYLD to TRAF6 to promote the LPA2 receptor-mediated TRAF6 activation

**DOI:** 10.1038/celldisc.2015.48

**Published:** 2016-03-01

**Authors:** Fang-Tsyr Lin, Vivian Y Lin, Victor T G Lin, Weei-Chin Lin

**Affiliations:** 1 Department of Medicine, Section of Hematology/Oncology, Baylor College of Medicine, Houston, TX, USA; 2 Department of Cell Biology, University of Alabama at Birmingham, Alabama, AL, USA

**Keywords:** TRAF6, TRIP6, NF-κB, LPA, A20, CYLD, JNK

## Abstract

The elevated lysophosphatidic acid signaling has been causally linked to cancer-associated inflammation and tumorigenesis through upregulation of nuclear factor-κB signaling. However, how this signaling event is regulated has not yet been fully understood. Here we demonstrate that TRIP6, an LPA2 receptor-interacting adaptor protein, functions as a positive regulator of nuclear factor-κB and JNK signaling through direct binding to and activation of the E3 ligase TRAF6. Upon lysophosphatidic acid stimulation, TRIP6 recruits TRAF6 to the LPA2 receptor and promotes lysophosphatidic acid-induced JNK and nuclear factor-κB activation in a TRAF6-dependent manner. TRIP6 antagonizes the recruitment of deubiquitinases A20 and CYLD to TRAF6, thus sustaining the E3 ligase activity of TRAF6 and augmenting lysophosphatidic acid-activated nuclear factor-κB signaling. In contrast, depletion of TRIP6 by TRIP6-specific shRNA or Cas9/sgRNA greatly enhances the association of TRAF6 with A20 and CYLD, and attenuates lysophosphatidic acid-induced muclear factor-κB and JNK/p38 activation in ovarian cancer cells. On the other hand, TRAF6 also regulates TRIP6 by facilitating its binding to nuclear factor-κB p65 and phosphorylation by c-Src. Together, TRIP6 cooperates with TRAF6 to regulate the LPA2 receptor signaling, which may ultimately contribute to chronic inflammation, apoptotic resistance and cell invasion.

## Introduction

Lysophosphatidic acid (LPA) is a growth factor-like phospholipid that mediates cell proliferation, apoptotic resistance, angiogenesis and cancer invasion and metastasis via binding to the G protein-coupled LPA receptors [1]. LPA is also a potent proinflammatory mediator, which induces inflammatory responses through activation of nuclear factor κB (NF-κB), JNK and p38 pathways. It has been shown that LPA-induced NF-κB activation requires the CARMA3/MALT1/BCL10-mediated recruitment of the E3 ligase TRAF6 [2]. TRAF6 mediates K63-linked ubiquitination of NEMO/IKKγ, which in turn activates the IKK complex and phosphorylates IκB, leading to the degradation of IκB and allowing NF-κB to translocate to the nucleus for transcriptional regulation [3]. TRAF6 can also bind to the MAP3K family members, such as MEKK1, ASK1 or TAK1, to promote JNK and p38 MAP kinase signaling pathways [4–6], which in turn activates the downstream activator protein-1 (AP-1) transcriptional activity to regulate cell proliferation, differentiation and other cellular responses. By catalyzing K63-linked ubiquitination of AKT at its PH domain, TRAF6 has been shown to promote the membrane translocation and activation of AKT [7]. These findings suggest that TRAF6 functions to integrate multiple signaling pathways involved in inflammation, antiapoptosis and cell proliferation.

The NF-κB signaling is tightly controlled by negative feedback regulatory mechanisms. Once activated, NF-κB upregulates its own inhibitors, including IκBα, A20 and CYLD, to terminate the NF-κB signaling [8]. A20 and CYLD are deubiquitinating enzymes that remove the K63-specific ubiquitin chains from TRAF6 to restrict its activity. This negative feedback loop regulation is required to ensure the proper control of NF-κB activity since depletion of A20 or CYLD results in persistent NF-κB activation and inflammation [9, 10]. However, in many types of cancer, NF-κB is constitutively activated and induces expression of genes that promote cancer cell proliferation, invasion, angiogenesis and apoptotic resistance [11], suggesting that in cancers, either the proinflammatory mediators are aberrantly elevated and override the negative feedback control and/or mechanisms to terminate NF-κB activity are defective, thus enabling NF-κB to promote tumorigenesis.

Among the different LPA receptors, the LPA2 receptor has been found to be upregulated in various malignancies, such as ovarian cancer, colorectal cancer, gastric cancer, thyroid cancer and breast cancer [12–15]. Although overproduction of LPA and upregulation of the LPA2 receptor have been causally linked to cancer-associated inflammation and tumorigenesis in ovarian and breast cancers [16, 17]; thus far, it is not fully understood how TRAF6 is linked to the LPA2 receptor, and whether the function of A20 or CYLD in restricting LPA-stimulated NF-κB signaling is impaired in these cancers. In this regard, here we provide evidence that the adaptor protein TRIP6 (thyroid hormone receptor-interacting protein 6), a specific interacting protein of the LPA2 receptor but not other LPA receptors, recruits TRAF6 to the LPA2 receptor and enhances the E3 ligase activity of TRAF6 by antagonizing the association of A20 and CYLD to TRAF6.

Our earlier studies show that TRIP6 binds to a unique CXXC motif of the LPA2 receptor to regulate its function in cell motility and antiapoptotic responses [18, 19]. TRIP6 functions at a point of convergence of multiple signaling pathways critical for cancer development, including c-Src/ERK, PI3K/AKT and NF-κB. It is highly expressed in several types of cancer, such as glioblastoma, colon cancer and ovarian cancer [20–23]. Through the three LIM domains, a PDZ-binding motif, a Crk SH2-binding motif and several putative SH3-binding domains, TRIP6 serves as a platform to recruit a number of molecules involved in cell motility, cell proliferation and antiapoptotic signaling [22].

Independent studies show that TRIP6 regulates NF-κB at multiple levels. In addition to serving as a coactivator of nuclear NF-κB p65 in transcriptional control [24, 25], TRIP6 can bind to NF-κB p65 in the cytosol and promote its nuclear translocation upon LPA stimulation or Fas/CD95 activation [21]. Moreover, TRIP6 is found to be present in the RIP2-associated multiprotein complex to potentiate NF-κB activation by tumor necrosis factor-α (TNF-α), IL-1, Nod1 and TLR2 [26]. These findings suggest that TRIP6 may function as a further upstream regulator of NF-κB signaling. Indeed, our new data show that TRIP6 associates with TRAF6 directly, and recruits TRAF6 to the LPA2 receptor to promote LPA-stimulated NF-κB and JNK signaling. Our data suggest a reciprocally positive regulation between TRIP6 and TRAF6 in the LPA2 receptor signaling, which may ultimately contribute to chronic inflammation, apoptotic resistance and tumorigenesis.

## Results

### Depletion of TRIP6 greatly attenuates LPA-induced NF-κB and JNK/p38 activation in ovarian cancer cells

**Figure 1:**
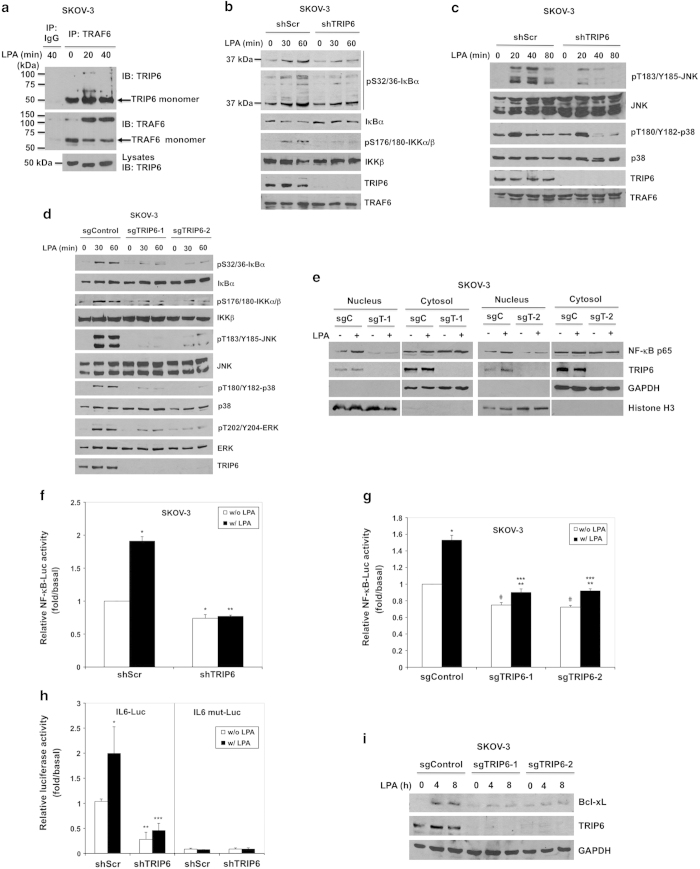
Depletion of TRIP6 eliminates LPA-induced NF-κB and MAP kinase activation in ovarian cancer cells. (**a**) TRIP6 associates with TRAF6 constitutively in SKOV-3 cells. SKOV-3 cells were starved for 24 h, followed by stimulation with LPA for 20 or 40 min. Endogenous TRAF6 was immunoprecipitated with anti-TRAF6 mouse monoclonal antibody or control mouse IgG, followed by immunoblotting with anti-TRIP6 rabbit antibody. The blot was reprobed with anti-TRAF6 rabbit antibody. The bottom panel shows the expression of endogenous TRIP6 in the whole-cell lysates. (**b**) Knockdown of TRIP6 attenuates LPA-induced IKK activation and IκBα phosphorylation. SKOV-3 cells stably expressing scrambled shRNA or TRIP6 shRNA were starved overnight, followed by stimulation with LPA for 30 or 60 min. Immunoblotting was performed to determine the levels of phospho-S32/36-IκBα, phospho-S176/180-IKKα/β, total IκBα, IKKβ, TRIP6 or TRAF6 in the whole-cell lysates. (**c**) Knockdown of TRIP6 reduces LPA-induced JNK activation. SKOV-3 cells stably expressing scrambled shRNA or TRIP6 shRNA were starved overnight, and then treated with LPA for 20, 40 or 80 min. Immunoblotting was performed to detect phosphorylated or total JNK, p38, TRIP6 or TRAF6 in the whole-cell lysates. (**d**) Depletion of TRIP6 by CRISPR/Cas9/sgRNA-mediated targeting of TRIP6 gene greatly eliminates LPA-stimulated IκBα phosphorylation and MAP kinase activation. SKOV-3 stable cell lines harboring Cas9 alone (sgControl) or Cas9/TRIP6 sgRNA (sgTRIP6-1, sgTRIP6-2) were treated with LPA for 30 or 60 min. Immunoblotting was performed to determine the levels of phosphorylated or total IκBα, IKKα/β, JNK, p38, ERK or TRIP6 in the whole-cell lysates. (**e**) Depletion of TRIP6 reduces LPA-promoted nuclear translocation of NF-κB p65. SKOV-3 cells harboring Cas9 alone (sgC) or Cas9/TRIP6 sgRNA (sgT-1, sgT-2) were starved overnight, followed by LPA stimulation for 90 min. Subcellular fractionation was performed in hypotonic buffer to separate the nucleus (pellet) from the cytosol (supernatant). The protein levels of NF-κB p65 or TRIP6 in each fraction were determined by immunoblotting. Glyceraldehyde-3-phosphate dehydrogenase (GAPDH) and Histone H3 serve as cytosolic and nuclear markers, respectively. Data shown in (**a**–**e**) are representative of two to four independent experiments. (**f** and **g**) Depletion of TRIP6 reduces the basal and LPA-promoted NF-κB activity in SKOV-3 cells. SKOV-3 cells stably expressing shRNA (shScr, shTRIP6) (**f**) or Cas9/sgRNA (sgControl, sgTRIP6-1, sgTRIP6-2) (**g**) were transfected with the expression vectors of NF-κB-Luc and β-galactosidase. Cells were starved for 24 h, followed by LPA stimulation for 3 h. The NF-κB-driven luciferase activity was measured and normalized to the β-galactosidase activity. Data shown in (**f**) are the mean±s.e.m. of six independent experiments (*P<0.001 versus untreated shScr cells; **P<0.001 versus treated shScr cells; Student’s *t-*test). Data shown in (**g**) are the mean±s.e.m. of four independent experiments (*P<0.001, **P<0.05 versus untreated cells; ***P<0.001 versus treated sgControl cells; ^#^*P*<0.001 versus untreated sgControl cells; Student’s *t-*test). (**h**) Knockdown of TRIP6 attenuates IL-6 activity in SKOV-3 cells. SKOV-3 cells stably expressing scrambled shRNA or TRIP6 shRNA were transfected with the expression vector of β-galactosidase and either pIL-6-Luc or pIL-6 mut-Luc with mutation in the NF-κB binding site. After stimulation for 3 h, the IL-6-driven luciferase activity was measured and normalized to the β-galactosidase activity. Data shown are the mean±s.e.m. of three independent experiments carried out in duplicates or triplicates (*P<0.05, **P<0.01 versus untreated shScr cells; ***P<0.01 versus treated shScr cells; Student’s *t-*test). (**i**) Depletion of TRIP6 attenuates LPA-induced Bcl-xL expression. SKOV-3 cells stably expressing Cas9 alone (sgControl) or Cas9/TRIP6 sgRNA (sgTRIP6-1, sgTRIP6-2) were starved overnight, followed by LPA stimulation for 4 or 8 h. Immunoblotting was performed to detect the levels of Bcl-xL, TRIP6 or GAPDH. The result shown is representative of two independent experiments.

Although TRIP6 has been shown to regulate the NF-κB signaling by serving as a coactivator of NF-κB p65 in the nucleus [25], our previous data show that knockdown of TRIP6 increases the association of IκBα with NF-κB p65 in the cytosol and attenuates LPA-induced nuclear translocation of NF-κB p65 [21], suggesting that TRIP6 may function as a further upstream regulator of NF-κB signaling. As TRIP6 contains a consensus TRAF6-binding motif (PXEXXAr/Ac) in its N-terminal region, we thought to investigate whether TRIP6 promotes LPA-induced NF-κB activation through binding to and activation of TRAF6. Indeed, under physiological conditions, TRIP6 bound to TRAF6 constitutively in SKOV-3 ovarian cancer cells that express TRIP6 and the LPA2 receptor at high levels (Figure 1a). At some conditions when the protein complexes were not completely resolved by sodium dodecyl sulfate polyacrylamide gel electrophoresis, we also observed several dimerized and modified high-molecular-weight forms of TRAF6 and TRIP6 in the complexes upon LPA stimulation, implicating an oligomeric complex formation between TRIP6 and TRAF6.

To determine if TRIP6 affects the TRAF6 activity, we first examined the effect of TRIP6 depletion on LPA-induced activation of IKK complex and JNK/p38 in SKOV-3 cells. We found that knockdown of TRIP6 by its specific shRNA attenuated LPA-stimulated IKKα/β activation and IκBα phosphorylation (Figure 1b). The multiple high-molecular-weight forms of phospho-IκBα revealed by longer exposure of the immunoblot are presumably polyubiquitinated phospho-IκBα. Knockdown of TRIP6 also reduced LPA-induced JNK activation (Figure 1c). Although TRIP6 knockdown did not affect the early induction of p38 activity by LPA, it indeed decreased the levels of activated p38 at 40–80 min of treatment. To further confirm these phenotypes, we performed CRISPR/Cas9/sgRNA technology to specifically target exon 2 of TRIP6 gene in SKOV-3 cells. The result showed that LPA-induced IκBα phosphorylation and activation of IKKα/β, JNK, p38 and ERK were greatly reduced in two SKOV-3 clonal cell lines (sgTRIP6-1, sgTRIP6-2) that showed the highest efficiency of TRIP6 knockout (Figure 1d). Moreover, disruption of TRIP6 gene by Cas9/sgRNA significantly decreased the levels of nuclear NF-κB p65 in the absence or presence of LPA (Figure 1e). Perhaps because the basal NF-κB activity is already high in SKOV-3 cells, the luciferase reporter assay showed that LPA only modestly, but significantly elevated the NF-κB activity in these cancer cells (Figure 1f). Nonetheless, depletion of TRIP6 by either TRIP6-specific shRNA (Figure 1f) or Cas9/sgRNA (Figure 1g) not only reduced the basal NF-κB activity, but also LPA-promoted activation of NF-κB.

LPA has been shown to promote the IL-6 expression through activation of NF-κB and AP-1 [27]. Indeed, LPA stimulation enhanced the IL-6 promoter-driven luciferase expression in SKOV-3 cells, but this effect was abolished by mutation of the NF-κB-binding site in the IL-6 promoter (Figure 1h). When TRIP6 was knocked down, this significantly reduced the basal and LPA-promoted IL-6 activity, confirming a regulatory role for TRIP6 in NF-κB-activated IL-6 expression. LPA has also been shown to activate the antiapoptotic protein Bcl-xL [28], a transcriptional target of NF-κB and AP-1 [29, 30] Consistently, our data showed that depletion of TRIP6 by Cas9/sgRNA reduced LPA-promoted expression of Bcl-xL in SKOV-3 cells (Figure 1i).

To determine if TRIP6 plays a specific role in LPA-stimulated NF-κB and JNK signaling, we also examined the effect of TRIP6 on TNF-α-induced NF-κB and JNK activation. The result showed that depletion of TRIP6 by either TRIP6 shRNA (Supplementary Figure S1a) or Cas9/TRIP6 sgRNA (Supplementary Figure S1b) only mildly or barely reduced TNF-α-induced IκBα phosphorylation and JNK activation in SKOV-3 cells, suggesting that TRIP6 does not have significant impact on TNF-α signaling in these cells. Taken together, these findings indicate a significant role for TRIP6 in LPA-stimulated NF-κB and JNK/p38 signaling in SKOV-3 ovarian cancer cells.

### TRIP6 recruits TRAF6 to the LPA2 receptor and promotes the LPA2 receptor-mediated NF-κB and JNK activation in a TRAF6-dependent manner

**Figure 2:**
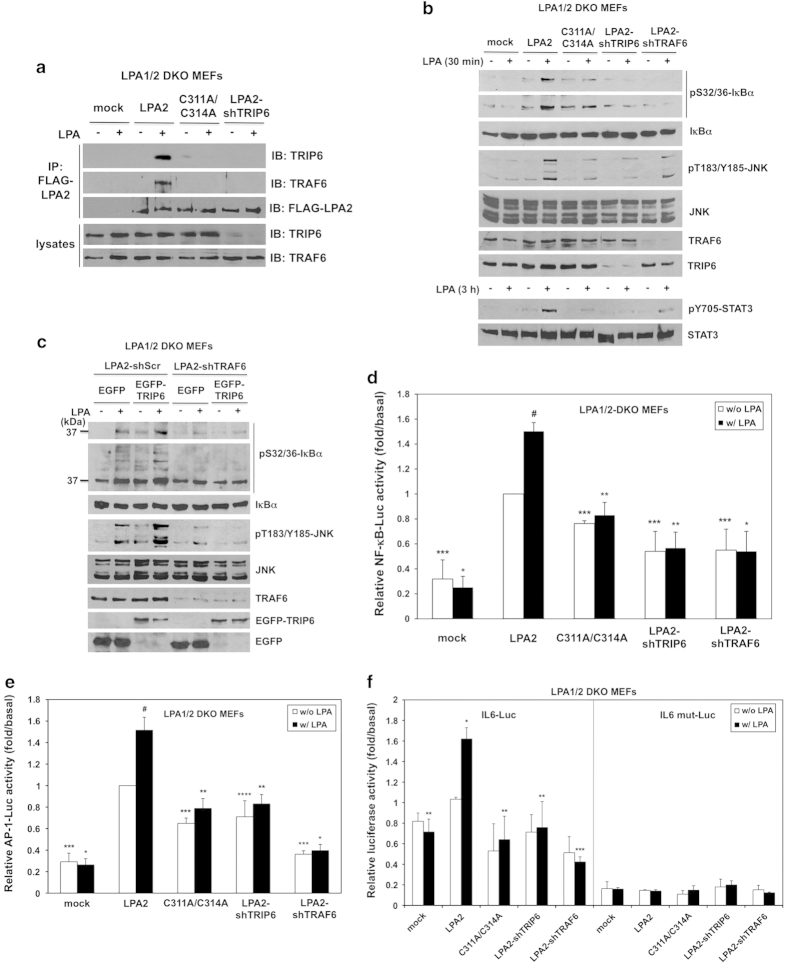
TRIP6 recruits TRAF6 to the LPA2 receptor and promotes the LPA2 receptor-mediated JNK and NF-κB activation in a TRAF6-dependent manner. (**a**) Disruption of the LPA2 receptor binding to TRIP6 or knockdown of TRIP6 expression eliminates LPA-induced association of TRAF6 with the LPA2 receptor. The immortalized LPA1/2 DKO MEFs stably harboring an empty vector (mock), wild-type or C311A/C314A FLAG-LPA2 receptor, or FLAG-LPA2 receptor with mouse TRIP6 shRNA (shTRIP6) were starved for 5 h, followed by stimulation with 2 μm LPA for 30 min. The FLAG-LPA2 receptor was immunoprecipitated with anti-FLAG M2 mouse monoclonal antibody-conjugated agarose beads, followed by immunoblotting with antibody specific to TRIP6, TRAF6 or FLAG epitope to determine the presence of endogenous TRIP6 or TRAF6 in the FLAG-LPA2 receptor complex. The bottom two panels show the expression of endogenous TRIP6 and TRAF6 in the whole-cell lysates. (**b**) Disruption of the LPA2 receptor binding to TRIP6 or knockdown of TRIP6 or TRAF6 attenuates LPA-induced IκBα phosphoylation and JNK activation. The immortalized LPA1/2 DKO MEFs stably harboring an empty vector (mock), wild-type or C311A/C314A FLAG-LPA2 receptor, or FLAG-LPA2 receptor with either mouse TRIP6 shRNA (shTRIP6) or mouse TRAF6 shRNA (shTRAF6) were starved for 5 h, followed by treatment with 2 μm LPA for 30 min or 3 h. Immunoblotting was performed to determine the levels of phosphorylated or total IκBα, JNK, STAT3, TRIP6 or TRAF6 in the whole-cell lysates. (**c**) TRIP6 regulates the LPA2 receptor-mediated IκBα phosphorylation and JNK activation in a TRAF6-dependent manner. The LPA1/2 DKO MEFs stably expressing FLAG-LPA2 receptor with either scrambled shRNA or TRAF6 shRNA were transduced with lentivirus harboring either EGFP or EGFP-TRIP6. Cells were starved for 5 h, followed by LPA stimulation for 30 min. Immunoblotting was performed to determine the levels of phosphorylated or total IκBα, JNK, TRAF6, EGFP-TRIP6 or EGFP in the whole-cell lysates. Data shown in (**a**–**c**) are representative of three independent experiments. (**d**–**f**) Disruption of the LPA2 receptor binding to TRIP6 or knockdown of either TRIP6 or TRAF6 reduces the LPA2 receptor-mediated NF-κB and AP-1 activation. The LPA1/2-DKO MEF stable cell lines as indicated were transiently transfected with the expression vectors of β-galactosidase and either NF-κB-Luc (**d**), AP-1-Luc (**e**), IL-6-Luc or IL-6 mut-Luc with mutation in the NF-κB-binding site (**f**). After starvation for 5 h, cells were treated with LPA for another 3 h. Luciferase activity was measured and normalized to the β-galactosidase activity. In (**d**), data shown are the mean±s.e.m. of four independent experiments (*P<0.01, **P<0.05 versus treated LPA2 MEFs; ***P<0.01, ^#^*P*<0.001 versus untreated LPA2 MEFs; Student’s *t-test*). In (**e**), data shown are the mean±s.e.m. of five independent experiments (*P<0.001, **P<0.01 versus treated LPA2 MEFs; ***P<0.001, ****P<0.05, ^#^*P*<0.01 versus untreated LPA2 MEFs; Student’s *t-test*). In (**f**), data shown are the mean±s.e.m. of three independent experiments carried out in triplicates (*P<0.01 versus untreated LPA2 MEFs; **P<0.05, ***P<0.01 versus treated LPA2 MEFs; Student’s *t-*test).

Among the different LPA receptors, the LPA2 receptor is recognized as the most efficient one that mediates the production of IL-6 and IL-8 in ovarian cancer cells through activation of NF-κB and AP-1 [27]. IL-6 and IL-8 can further activate STAT3, which has been shown to promote apoptotic resistance and contribute to poor prognosis of cancer patients [31, 32] Although TRAF6 is known to mediate LPA-stimulated NF-κB activation [2], it is not clear whether TRAF6 plays a particular role in the LPA2 receptor signaling. As the adaptor protein TRIP6 not only associates with TRAF6 (Figure 1a), but also binds directly to a ^311^CXXC motif located at the carboxyl-terminal tail of the LPA2 receptor [18], it is likely that TRIP6 plays a specific role in recruiting TRAF6 to the LPA2 receptor. To address this possibility, we first asked if LPA stimulation induces the association of TRAF6 with the LPA2 receptor, and whether disruption of the LPA2 receptor binding to TRIP6 by the C311A/C314A mutation [18] or depletion of TRIP6 could affect this interaction. Thus, wild type or C311A/C314A mutant FLAG-LPA2 receptor was expressed at comparable levels in the immortalized *LPA1*^*−/−*^*, LPA2*^*−/−*^ mouse embryonic fibroblasts (LPA1/2 DKO MEFs) (Figure 2a). The LPA1/2 DKO MEFs stably expressing FLAG-LPA2 receptor were further transduced with lentivirus harboring a mouse TRIP6-specific shRNA (shTRIP6). Subcellular fractionation confirmed that disruption of the LPA2 receptor binding to TRIP6 by the C311A/C314A mutation or knockdown of TRIP6 did not impair the expression of LPA2 receptor on the plasma membrane (Supplementary Figure S2). Under this condition, LPA stimulation for 30 min induced the association of both TRIP6 and TRAF6 with the FLAG-LPA2 receptor; however, these interactions were abolished by the C311A/C314A mutation of LPA2 receptor, or knockdown of TRIP6 expression (Figure 2a), indicating a specific role for TRIP6 in this regulation.

We next examined the effect of TRIP6 on the LPA2 receptor-mediated IκBα phosphorylation and JNK activation. By LPA stimulation for 30 min, we observed that knockout of both LPA1 and LPA2 receptors completely abolished LPA-induced IκBα phosphorylation or JNK activation; however, these effects could be restored by stable expression of FLAG-LPA2 receptor in these MEFs (Figure 2b). Nonetheless, disruption of the LPA2 receptor binding to TRIP6 by C311A/C314A mutation or knockdown of either TRIP6 or TRAF6 greatly attenuated LPA-induced IκBα phosphorylation and JNK activation (Figure 2b). Accordingly, prolonged LPA-stimulated STAT3 activation was also significantly reduced by C311A/C314A mutation of the LPA2 receptor or knockdown of TRIP6 or TRAF6.

To determine if TRIP6 regulates the LPA2 receptor-mediated IκBα phosphorylation and JNK activation through TRAF6, we next asked whether the effect of TRIP6 on promoting LPA-induced IκBα phosphorylation and JNK activation could be blocked by TRAF6 knockdown in the LPA1/2 DKO MEFs stably expressing FLAG-LPA2 receptor. Indeed, overexpression of EGFP-TRIP6 was able to enhance LPA-induced IκBα phosphorylation and JNK activation; however, this effect was abolished by TRAF6 knockdown (Figure 2c). This result indicates that TRIP6 promotes the LPA2 receptor-mediated activation of NF-κB and JNK signaling through TRAF6-dependent mechanisms. Consistent with these findings, the luciferase reporter assays showed that stable expression of FLAG-LPA2 receptor in the LPA1/2 DKO MEFs greatly elevated the basal transcriptional activity of NF-κB (Figure 2d) or AP-1 (Figure 2e); however, these effects were attenuated by either C311A/C314A mutation of the LPA2 receptor, or knockdown of TRIP6 or TRAF6. Under this condition, addition of LPA for 3 h further modestly, but significantly increased the activity of NF-κB (Figure 2d) or AP-1 (Figure 2e) in the LPA1/2 DKO MEFs stably expressing FLAG-LPA2 receptor, but not those expressing C311A/C314A mutant, or FLAG-LPA2 receptor with TRIP6 shRNA or TRAF6 shRNA. Accordingly, LPA stimulation increased the luciferase expression driven by the IL-6 promoter, but not that lacking the NF-κB-binding site, in the LPA1/2 DKO MEFs stably expressing FLAG-LPA2 receptor (Figure 2f). Nonetheless, disruption of the LPA2 receptor binding to TRIP6 by the C311A/C314A mutation or knockdown of TRIP6 or TRAF6 significantly reduced LPA-promoted IL-6 activation (Figure 2f). Together, these results suggest that TRIP6 recruits TRAF6 to the LPA2 receptor and promotes LPA-stimulated NF-κB and JNK-AP-1 activation in a TRAF6-dependent manner.

### Overexpression of TRIP6 interferes with the recruitment of A20 to TRAF6, whereas depletion of TRIP6 enhances the association of TRAF6 with A20 and CYLD, and eliminates LPA-promoted K63-linked polyubiquitination of TRAF6

**Figure 3:**
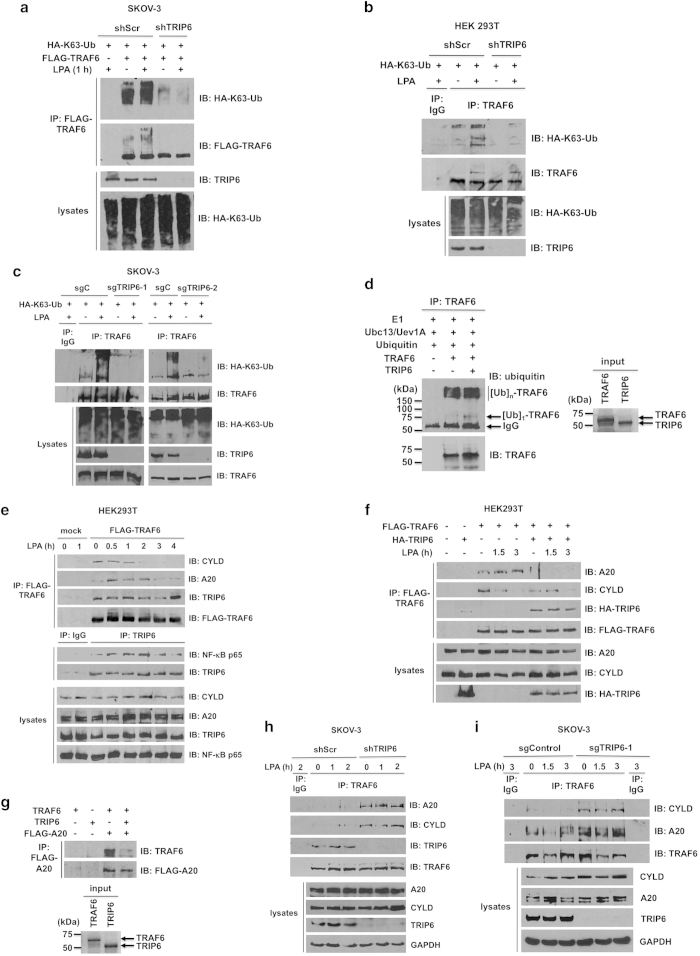
TRIP6 regulates LPA-induced K63-linked polyubiquitination of TRAF6, and antagonizes the recruitment of A20 and CYLD to TRAF6. (**a**) Knockdown of TRIP6 attenuates K63-linked polyubiquitination of transfected TRAF6. SKOV-3 cells stably expressing scrambled shRNA or TRIP6 shRNA were transfected with the expression vectors of HA-K63-ubiquitin and FLAG-TRAF6. Cells were starved for 6 h, followed by LPA stimulation for 1 h. Heat-denatured FLAG-TRAF6 was immunoprecipitated with anti-FLAG M2 monoclonal antibody-conjugated agarose beads, followed by immunoblotting using anti-HA antibody to detect K63-linked polyubiquitinated FLAG-TRAF6. After stripping, the immunoblot was reprobed with anti-FLAG antibody to detect immunoprecipitated FLAG-TRAF6. The bottom two panels show the expression of total HA-K63-ubiquitin and TRIP6 in the whole-cell lysates. (**b** and **c**) Depletion of TRIP6 eliminates LPA-stimulated K63-linked polyubiquitination of endogenous TRAF6. HEK293T cells stably harboring scrambled shRNA or TRIP6 shRNA (**b**), or SKOV-3 cells stably harboring Cas9 alone (sgC) or Cas9/TRIP6 sgRNA (sgTRIP6-1, sgTRIP6-2) (**c**) were transfected with the HA-K63-ubiquitin expression vector. Cells were starved overnight, followed by stimulation with LPA for 1 h. Heat-denatured endogenous TRAF6 was immunoprecipitated with anti-TRAF6 mouse monoclonal antibody, followed by immunoblotting with anti-HA antibody to detect K63-linked polyubiquitinated TRAF6. The immunoblot was reprobed with anti-TRAF6 rabbit polyclonal antibody to detect immunoprecipitated TRAF6. (**d**) TRIP6 barely enhances the *in vitro* autoubiquitination of TRAF6. The *in vitro* ubiquitination assay was performed by incubating E1, Ubc13/Uev1A (E2), ubiquitin and ATP without or with purified recombinant TRAF6 and TRIP6 at 30 °C for 1 h. After heat denaturation, TRAF6 was immunoprecipitated with anti-TRAF6 mouse monoclonal antibody, followed by immunoblotting with anti-ubiquitin rabbit antibody to detect autoubiquitinated TRAF6. The blot was reprobed with anti-TRAF6 rabbit antibody. The right panel shows coomassie blue staining of purified TRAF6 and TRIP6 used in this experiment. (**e**) LPA stimulation decreases the association of TRAF6 with CYLD, but promotes its binding to A20, whereas TRIP6 binds to TRAF6 constitutively and associates with NF-κB p65 following LPA stimulation. HEK293T cells expressing FLAG-TRAF6 were starved for 6 h, followed by LPA stimulation for the indicated times. FLAG-TRAF6 was immunoprecipitated with anti-FLAG M2 mouse monoclonal antibody-conjugated agarose beads, and co-immunoprecipitated endogenous CYLD, A20 or TRIP6 was detected by immunoblotting using rabbit antibody specific to each protein. Endogenous TRIP6 was immunoprecipitated with anti-TRIP6 mouse monoclonal antibody or control mouse IgG, followed by immunoblotting to detect co-immunoprecipitated endogenous NF-κB p65. The bottom four panels show the expression of endogenous CYLD, A20, TRIP6 or NF-κB p65 in the whole-cell lysates. (**f**) Overexpression of TRIP6 eliminates LPA-promoted interaction between TRAF6 and A20, and mildly attenuates the association of TRAF6 with CYLD in the absence of LPA. HEK293T cells expressing FLAG-TRAF6 alone or with HA-TRIP6 were starved for 6 h, followed by stimulation with LPA for 1.5 or 3 h. FLAG-TRAF6 was immunoprecipitated with anti-FLAG M2 mouse monoclonal antibody-conjugated agarose beads. Co-immunoprecipitated A20, CYLD or HA-TRIP6 was detected by immunoblotting using rabbit antibody specific to A20, CYLD or HA epitope. (**g**) TRIP6 physically interferes with the binding of TRAF6 to A20. HEK293T cells harboring an empty vector or FLAG-A20 were treated with LPA for 1.5 h, and then harvested in RIPA buffer. FLAG-A20 was immunoprecipitated with anti-FLAG M2 mouse monoclonal antibody-conjugated agarose beads. The beads were then aliquoted and incubated with 0.1 μg purified recombinant TRAF6 in the absence or presence of 0.1 μg purified recombinant TRIP6 at 4 °C for 3 h. After washing four times, the beads were subjected to immunoblotting with anti-TRAF6 rabbit antibody to detect co-immunoprecipitated recombinant TRAF6. The blot was stripped and reprobed with anti-FLAG antibody to detect immunoprecipitated FLAG-A20. The right panel shows coomassie blue staining of purified recombinant TRAF6 and TRIP6. (**h** and **i**) Depletion of TRIP6 greatly enhances the binding of TRAF6 to A20 and CYLD in SKOV-3 cells. SKOV-3 cell lines stably expressing shRNA (shScr, shTRIP6) (**h**) or Cas9/sgRNA (sgControl, sgTRIP6-1) (**i**) were starved overnight, followed by LPA treatment for various times as indicated. Endogenous TRAF6 was immunoprecipitated with anti-TRAF6 mouse monoclonal antibody or control mouse IgG, followed by immunoblotting with rabbit antibody specific to A20, CYLD, TRIP6 or TRAF6. The bottom four panels in each figure show the expression of endogenous A20, CYLD, TRIP6 or GAPDH in the whole-cell lysates. Data shown in each figure are representative of two to four independent experiments.

To understand how TRIP6 regulates TRAF6 activity, we first examined the effect of TRIP6 depletion on the K63-linked autoubiquitination of TRAF6. Using shRNA-mediated knockdown of TRIP6 in SKOV-3 (Figure 3a) or HEK293T cells (Figure 3b) as well as Cas9/sgRNA-directed knockout of TRIP6 (Figure 3c), we found that depletion of TRIP6 greatly eliminated LPA-promoted K63-linked polyubiquitination of TRAF6. We next examined if TRIP6 affects the autoubiquitination of TRAF6 directly. However, the *in vitro* ubiquitination assay showed that autoubiquitination of purified recombinant TRAF6 was barely or only slightly enhanced by adding purified TRIP6 (Figure 3d), suggesting that TRIP6 may regulate TRAF6 activity through other mechanisms. This finding prompted us to investigate whether TRIP6 regulates the TRAF6 activity by interfering with the recruitment of its deubiquitinases, such as A20 or CYLD.

Thus far, very little is known about how the deubiquitinating enzyme A20 or CYLD is regulated during LPA signaling. To address this issue, we expressed FLAG-TRAF6 in HEK293T cells and treated cells with LPA for various times to determine how TRAF6 associates with deubiquitinase A20 or CYLD. The data showed that CYLD bound to TRAF6 constitutively, but this interaction was gradually diminished following prolonged LPA stimulation (Figure 3e). On the other hand, A20 mildly associated with TRAF6 under starved conditions, and this interaction was transiently enhanced by LPA until 3 h of treatment when the binding started to decline. In contrast, TRIP6 bound to TRAF6 constitutively, and this binding was decreased at 2 h of LPA treatment when the association of TRIP6 with NF-κB p65 reached the peak levels. It appeared that TRIP6 and TRAF6 re-associated by 4-h treatment.

We next examined whether TRIP6 affects the recruitment of either A20 or CYLD to TRAF6 during LPA treatment in HEK293T cells. We found that the association of A20 with transfected TRAF6 was greatly eliminated in untreated and treated cells by overexpression of TRIP6 (Figure 3f). Perhaps because the levels of CYLD and the binding of CYLD to TRAF6 were already reduced following LPA stimulation, we only observed a reduction of this association by TRIP6 overexpression in unstimulated cells. These findings suggest that TRIP6 strongly antagonizes the binding of TRAF6 to A20, but has less impact on its association with CYLD in HEK293T cells.

Since we did not find a direct interaction between TRIP6 and A20, we speculated that TRIP6 might compete with A20 for the binding to TRAF6. To address this possibility, we purified FLAG-A20 from transfected HEK293T cells after LPA treatment for 90 min, and then assessed the *in vitro* binding between FLAG-A20 and purified recombinant TRAF6 in the absence or presence of recombinant TRIP6. The result showed that the interaction between TRAF6 and FLAG-A20 was significantly reduced when recombinant TRIP6 was added (Figure 3g), confirming that TRIP6 prevents the binding of A20 to TRAF6 through physical interference.

The persistent activation of NF-κB is frequently found in many types of cancers, as a result of elevated upstream signaling and/or reduction of negative regulatory control. Indeed, in contrast to HEK293T cells, SKOV-3 cells showed very weak association between TRAF6 and either A20 or CYLD in the absence or presence of LPA (Figure 3h and i). Nonetheless, depletion of TRIP6 by either shRNA (Figure 3h) or Cas9/sgRNA (Figure 3i) not only enhanced the association of TRAF6 with A20, but also with CYLD in untreated and treated cells, suggesting a significant role for TRIP6 in antagonizing the binding of TRAF6 to both A20 and CYLD in ovarian cancer cells. Likewise, in the U373-MG glioblastoma cells that showed constitutive NF-κB activity and high levels of TRIP6, depletion of TRIP6 by Cas9/sgRNA also attenuated the basal and LPA-stimulated IκBα phosphorylation and JNK activation (Supplementary Figure S3a). Moreover, TRAF6 barely associated with A20 without or with LPA stimulation in these cells; however, this binding could be enhanced by depletion of TRIP6 (Supplementary Figure S3b). Perhaps because U373-MG cells expressed very low levels of CYLD, we could barely detect the association of TRAF6 with CYLD even when TRIP6 was depleted. It may be noteworthy that the levels of CYLD were frequently, if not always, found to be slightly higher in U373-MG cells (Supplementary Figure S3b) or SKOV-3 cells (Figure 3i) stably expressing Cas9/TRIP6 sgRNA, and this might partly contribute to the effect of TRIP6 depletion on the reduced NF-κB activity in these cancer cells.

### The TRAF6-binding motif and LIM domains 1 to 2 of TRIP6 are responsible for its association with the carboxyl-terminal domain and RING domain of TRAF6, respectively, and are both required for its inhibition of A20 binding to TRAF6

**Figure 4:**
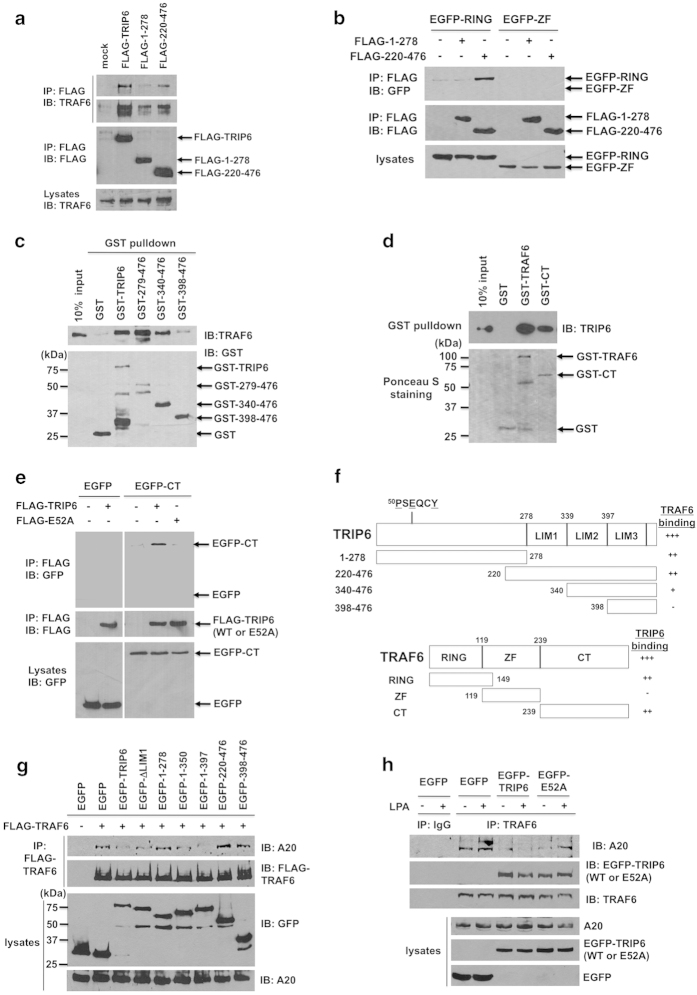
The TRAF6-binding motif and LIM domains 1 to 2 of TRIP6 are responsible for its interaction with the carboxyl-terminal domain and RING domain of TRAF6, respectively, and are both required for the blocking of A20 binding to TRAF6. (**a**) TRAF6 associates with the pre-LIM region and LIM domains of TRIP6. FLAG-TRIP6 or FLAG-TRIP6 mutant containing the pre-LIM region (residues 1–278) or three LIM domains (residues 220–476) was expressed in HEK 293T cells. FLAG proteins were immunoprecipitated with anti-FLAG M2 mouse monoclonal antibody-conjugated agarose beads, followed by immunoblotting with anti-TRAF6 antibody to detect co-immunoprecipitated endogenous TRAF6. The blot was reprobed with anti-FLAG antibody. The bottom panel shows the expression of endogenous TRAF6 in the whole-cell lysates. (**b**) The LIM domains of TRIP6 bind to the RING domain, but not zinc-finger domains, of TRAF6. FLAG-TRIP6 mutant containing the pre-LIM region (residues 1–278) or three LIM domains (residues 220–476) was co-expressed with an EGFP fusion protein containing either RING domain (residues 1–149) or zinc-finger (ZF) domains (residues 119–238) of TRAF6 in HEK293T cells. FLAG proteins were immunoprecipitated with anti-FLAG M2 mouse monoclonal antibody, followed by immunoblotting with anti-GFP antibody to detect co-immunoprecipitated EGFP fusion proteins. The blot was reprobed with anti-FLAG rabbit antibody. The bottom panel shows the expression of EGFP-tagged RING or zinc-finger domains of TRAF6 in the whole-cell lysates. (**c**) TRAF6 binds to LIM domains 1 to 2 of TRIP6 directly. Purified recombinant TRAF6 was incubated with GST, GST-TRIP6 or a GST-TRIP6 mutant containing either LIM domains 1 to 3 (residues 279–476), 2 to 3 (residues 340–476) or LIM3 domain (residues 398–476) at 4 °C for 3 h. TRAF6 pulled down by GST-TRIP6 or GST-TRIP6 mutant was detected by immunoblotting using anti-TRAF6 antibody. The bottom panel is an immunoblot of GST proteins detected with anti-GST antibody. (**d**) TRIP6 binds to the full-length TRAF6 or its carboxyl-terminal domain directly. Purified recombinant TRIP6 was incubated with GST, GST-TRAF6 or GST-CT mutant containing residues 239–530 of TRAF6 at 4 °C for 3 h. TRIP6 pulled down by GST-TRAF6 or GST-CT was detected by immunoblotting using anti-TRIP6 antibody. The bottom panel shows Ponceau S staining of GST fusion proteins. (**e**) The ability of TRIP6 to bind the carboxyl-terminal domain of TRAF6 is abolished by the E52A mutation. FLAG-TRIP6 or FLAG-E52A-TRIP6 was co-expressed with EGFP or EGFP-CT containing residues 239–530 of TRAF6 in HEK293T cells. Co-immunoprecipitation was performed as described in (**b**). Data shown in (**a**–**e**) are representative of two or three independent experiments. (**f**) Schematic structures of TRIP6 and TRAF6 and the domains responsible for their interactions. (**g**) The ability of TRIP6 to inhibit the association of TRAF6 with A20 requires both TRAF6-binding motif and LIM domains 1 to 2. HEK 293T cells transiently overexpressing FLAG-TRAF6 with either EGFP, EGFP-TRIP6, or one of the EGFP-TRIP6 deletion mutants were starved for 6 h, followed by addition of LPA for 90 min. Co-immunoprecipitation of FLAG-TRAF6 with endogenous A20 was performed as described in Figure 3e. The bottom two panels show the expression of EGFP, EGFP-tagged wild-type or mutant TRIP6, or A20 in the whole-cell lysates. (**h**) The ability of TRIP6 to block the recruitment of A20 to TRAF6 is reduced by the E52A mutation. HEK293T cells transiently expressing EGFP, EGFP-TRIP6 or EGFP-E52A-TRIP6 were starved for 6 h, and then treated with LPA for 90 min. Endogenous TRAF6 was immunoprecipitated with anti-TRAF6 mouse monoclonal antibody, followed by immunoblotting with anti-A20 or anti-GFP antibody to detect co-immunoprecipitated A20 or EGFP-TRIP6. The bottom three panels show the expression of A20, EGFP, EGFP-TRIP6 or EGFP-E52A-TRIP6 in the whole-cell lysates. Data shown in (**g**) and (**h**) are representative of three independent experiments.

We next performed structural analysis to determine how TRIP6 binds to TRAF6 and how this interaction affects the recruitment of A20 to TRAF6. To our surprise, the domain mapping showed that both the N-terminal pre-LIM region (residues 1–278) and C-terminal LIM domain-containing region (residues 220–476) of TRIP6 were able to bind TRAF6, albeit at a lower affinity than the full-length TRIP6 (Figure 4a). This result suggests that TRIP6 may associate with TRAF6 through multiple binding domains.

The structure of TRIP6 contains a consensus TRAF6-binding motif (PXEXXAr/Ac) in the N-terminal region and three LIM domains in the C-terminal region. The LIM domain is composed of two zinc fingers that structurally resemble the RING domain. Thus, we asked whether TRIP6 binds to the TRAF6-CT domain through its TRAF6-binding motif (^50^PSEQCY) and interacts with the RING domain or zinc-finger (ZF) domains of TRAF6 through its LIM domains. Indeed, deletion analysis confirmed that the LIM domains (residues 220–476) of TRIP6 bound to the RING domain, but not zinc-finger domains, of TRAF6; and the pre-LIM region (residues 1–278) of TRIP6 did not interact with either RING domain or zinc-finger domains of TRAF6 (Figure 4b). Since the *in vitro* GST pulldown assay showed that TRAF6 bound to the three LIM domains (residues 279–476) and to a lesser degree, to LIM domains 2 to 3 (residues 340–476), but did not interact with the LIM3 domain (residues 398–476) of TRIP6 (Figure 4c), we conclude that TRIP6 binds to the RING domain of TRAF6 through its LIM domains 1 to 2. On the other hand, although TRIP6 bound to the TRAF6-CT domain directly *in vitro* (Figure 4d), cellular co-immunoprecipitation showed that this association was disrupted by the E52A mutation of TRIP6 in HEK293T cells (Figure 4e), confirming that the TRAF6-binding motif (^50^PSEQCY) is required for TRIP6 to bind the TRAF6-CT domain. The binding domains responsible for the interaction between TRIP6 and TRAF6 are summarized in Figure 4f.

We next performed mutational analysis to determine how TRIP6 affects the association of TRAF6 with A20 in HEK293T cells. The data showed that the interaction of endogenous A20 with transfected FLAG-TRAF6 was eliminated by overexpression of EGFP-TRIP6 or EGFP-1–397 deletion mutant that contains both TRAF6-binding motif and LIM domains 1 to 2 of TRIP6 (Figure 4g). Meanwhile, EGFP-1–350 or EGFP-ΔLIM1 mutant that contains TRAF6-binding motif with either LIM1 or LIM2 domain could also partially inhibit the binding of TRAF6 to A20. In contrast, EGFP-1–278, 220–476 and 398–476 deletion mutants that contain the pre-LIM region, three LIM domains and LIM3 domain, respectively, failed to block this interaction. Likewise, overexpression of EGFP-TRIP6 abolished the association of endogenous TRAF6 with A20 in the absence or presence of LPA; however, this effect was attenuated by the E52A mutation (Figure 4h). Together, these results indicate that the function of TRIP6 in blocking the A20 binding to TRAF6 requires both TRAF6-binding motif and LIM domains 1 to 2.

### TRIP6 enhances TRAF6-NF-κB activity through the inhibition of A20 binding to TRAF6, but promotes LPA-stimulated TRAF6-JNK-AP-1 activation in an A20-independent manner

**Figure 5:**
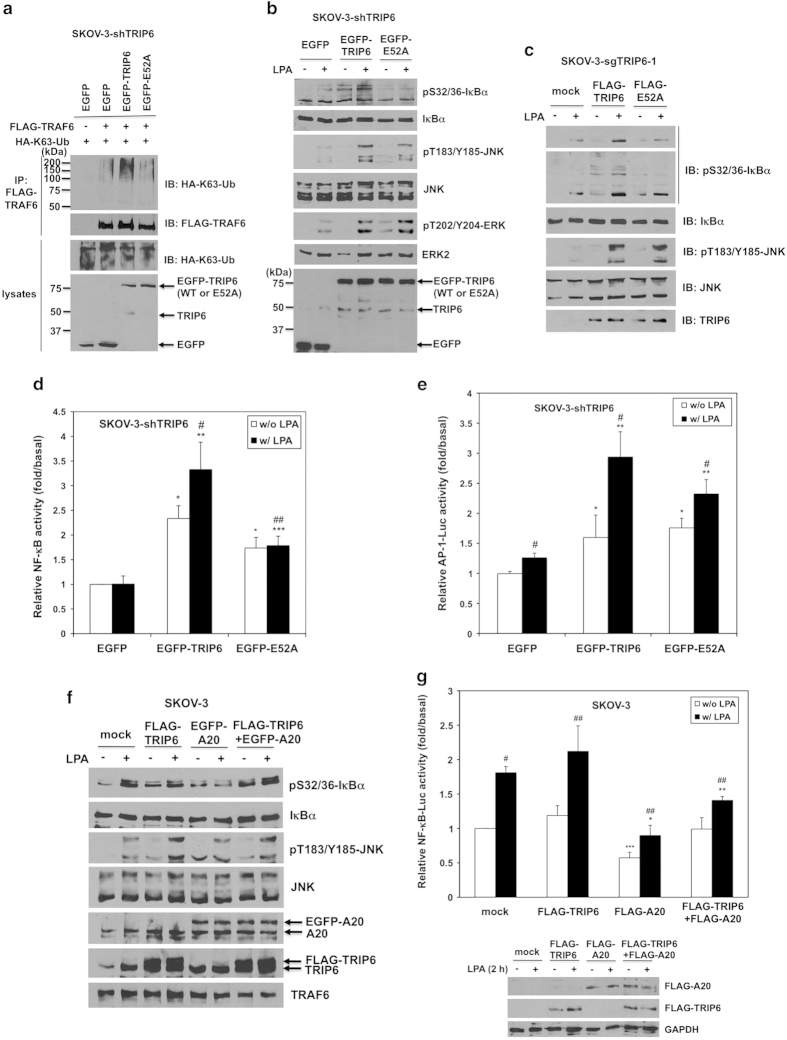
TRIP6 enhances TRAF6-NF-κB signaling by inhibiting the binding of A20 to TRAF6, but promotes LPA-induced TRAF6-JNK-AP-1 activation in an A20-independent manner. (**a**) The K63-linked polyubiquitination of TRAF6 is enhanced by wild-type TRIP6, but not E52A-TRIP6 mutant defective in blocking the recruitment of A20 to TRAF6. SKOV-3 cells stably expressing TRIP6 shRNA (SKOV-3-shTRIP6) were transiently transfected with the expression vectors of HA-K63-Ubiquitin, FLAG-TRAF6 and either EGFP, EGFP-TRIP6 or EGFP-E52A-TRIP6. After starvation for 24 h, cells were treated with LPA for 1 h. The levels of K63-linked polyubiquitinated FLAG-TRAF6 were determined as described in Figure 3a. The expression of HA-K63-ubiquitin, endogenous TRIP6, EGFP or EGFP-TRIP6 in the whole-cell lysates was detected by immunoblotting using antibody specific to HA, TRIP6 or GFP. (**b** and **c**) Mutation of E52 to Ala greatly eliminates the function of TRIP6 in promoting IκBα phosphorylation, but barely or only mildly attenuates its ability to promote LPA-induced JNK activation. SKOV-3 cells stably expressing TRIP6 shRNA (**b**) or Cas9/TRIP6 sgRNA (sgTRIP6-1) (**c**) were transiently transfected with EGFP- (**b**) or FLAG-tagged (**c**) wild-type or E52A TRIP6. Cells were starved for 24 h, followed by addition of LPA for 30 min. Immunoblotting was performed to detect the levels of indicated proteins in the whole-cell lysates. Data shown in (**a**–**c**) are representative of two or three independent experiments. (**d**) The effect of TRIP6 on promoting LPA-stimulated NF-κB activity is attenuated by the E52A mutation. SKOV-3-shTRIP6 cells were transiently transfected with pNF-κB-Luc, the β-galactosidase expression vector and either pEGFP, pEGFP-TRIP6 or pEGFP-E52A-TRIP6. Cells were starved for 24 h, followed by treatment with LPA for 3 h. The NF-κB-driven luciferase activity was determined and normalized to the β-galactosidase activity. Data shown are the mean±s.e.m. of four independent experiments done in duplicates (*P<0.001 versus untreated EGFP cells; **P<0.01, ***P<0.05 versus treated EGFP cells; ^#^*P*<0.05 versus untreated EGFP-TRIP6 cells; ^##^*P*<0.05 versus treated EGFP-TRIP6 cells; Student’s *t-test*). (**e**) The effect of TRIP6 on promoting LPA-induced AP-1 activity is not significantly affected by the E52A mutation. The expression vectors of AP-1-Luc and β-galactosidase were co-transfected with either pEGFP, pEGFP-TRIP6 or pEGFP-E52A-TRIP6 into SKOV-3-shTRIP6 cells. After starvation for 24 h, cells were treated with LPA for 3 h. The AP-1-driven luciferase activity was determined and normalized to the β-galactosidase activity. Data shown are the mean±s.e.m. of three independent experiments done in triplicates (*P<0.01 versus untreated EGFP cells; **P<0.001 versus treated EGFP cells; ^#^*P*<0.05 versus untreated cells; Student’s *t-test*). (**f**) A20 specifically inhibits LPA-stimulated IκBα phosphorylation, but not JNK activation; however, this effect is partially reversed by TRIP6 overexpression. SKOV-3 cells transiently overexpressing EGFP-A20 and/or FLAG-TRIP6 were starved in 0.1% fatty acid-free BSA-containing medium for 6 h, followed by stimulation with LPA for 30 min. Immunoblotting was performed to determine the levels of indicated proteins in the whole-cell lysates. Data shown are representative of two independent experiments. (**g**) The effect of A20 on the restriction of NF-κB activity is partially reversed by TRIP6 overexpression. NF-κB-Luc and β-galactosidase were transiently co-expressed with FLAG-A20 and/or FLAG-TRIP6 in SKOV-3 cells. Cells were starved overnight, followed by stimulation with LPA for 2 h. The NF-κB-driven luciferase activity was determined and normalized to the β-galactosidase activity. Data shown are the mean±s.e.m. of four independent experiments (*P<0.001, **P<0.05 versus treated mock cells; ***P<0.05 versus untreated mock cells; ^#^*P*<0.001, ^##^*P*<0.05 versus untreated cells; Student’s *t-test*). The bottom three panels are representative immunoblots showing the expression of FLAG-TRIP6, FLAG-A20 or GAPDH in the whole-cell lysates.

To further understand if TRIP6 promotes TRAF6 activity through the inhibition of A20 binding to TRAF6, we next examined if the function of TRIP6 in promoting LPA-stimulated TRAF6 activity could be affected by the E52A point mutant of TRIP6, which is defective in blocking the recruitment of A20 to TRAF6. Indeed, transiently adding EGFP-TRIP6 back to the TRIP6-depleted SKOV-3-shTRIP6 cells was able to enhance K63-linked polyubiquitination of TRAF6 (Figure 5a) as well as IκBα phosphorylation (Figure 5b); however, these effects were abolished by the E52A mutation. In contrast, the function of TRIP6 in promoting LPA-stimulated JNK activation was only mildly attenuated by the E52A mutation (Figure 5b). This is likely because the E52A mutant can still associate with TRAF6 through its LIM domains 1 to 2 and recruit it to the LPA2 receptor. On the other hand, the ability of TRIP6 to promote LPA-induced ERK activation was also not affected by the E52A mutation (Figure 5b). Consistently, the effect of TRIP6 depletion on the inhibition of LPA-induced IκBα phosphorylation could be rescued by transiently overexpressing wild-type TRIP6, but not E52A-TRIP6, whereas both wild-type and E52A TRIP6 were able to restore LPA-induced JNK activation comparably in the TRIP6-depleted SKOV-3-sgTRIP6-1 cells (Figure 5c). Accordingly, transient overexpression of EGFP-TRIP6 was able to enhance the basal and LPA-promoted NF-κB activity in the TRIP6-depleted SKOV-3-shTRIP6 cells; however, this effect was significantly reduced by the E52A mutation of TRIP6 (Figure 5d). Overexpression of EGFP-TRIP6 also promoted LPA-induced AP-1 activation in SKOV-3-shTRIP6 cells, and this effect was only mildly, but not significantly reduced by the E52A mutation (Figure 5e). Together, these findings indicate that the TRAF6-binding motif of TRIP6 is required for its function in the NF-κB signaling, but is dispensable for its effect on LPA-induced JNK activation. Consistent with this notion, overexpression of A20 did not affect LPA-induced JNK activation, but specifically blocked LPA-promoted IκBα phosphorylation (Figure 5f) and reduced the NF-κB activity without or with LPA treatment (Figure 5g). Although overexpression of TRIP6 did not further enhance IκBα phosphorylation (Figure 5f) or NF-κB activity (Figure 5g) in SKOV-3 cells, it increased LPA-induced JNK activation, and partially reversed the inhibitory effect of A20 on IκBα phosphorylation (Figure 5f) and NF-κB activity (Figure 5g). These results suggest that TRIP6 and A20 play opposing roles in TRAF6-mediated NF-κB signaling, whereas TRIP6 recruits TRAF6 to promote LPA-induced JNK activation in an A20-independent manner.

### The TRIP6–TRAF6–NF-κB signaling axis regulates the LPA2 receptor-mediated apoptotic resistance

**Figure 6:**
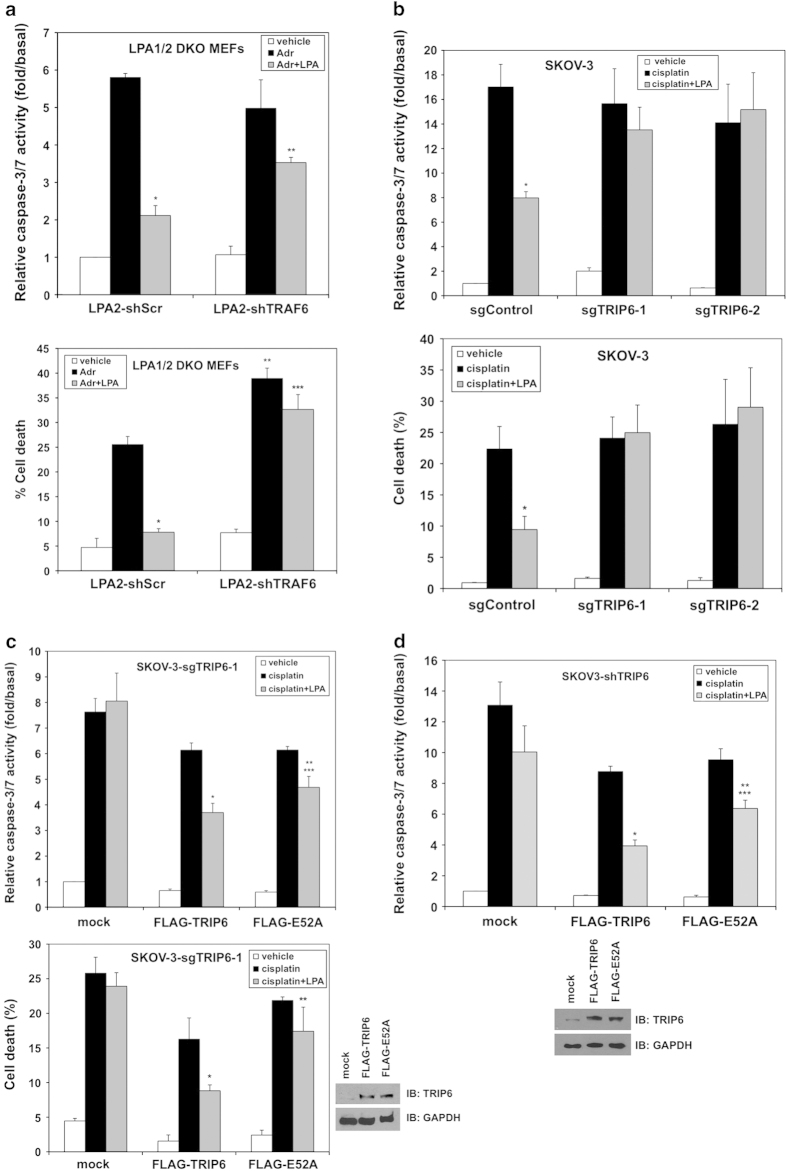
The function of TRIP6 in promoting the LPA2 receptor-mediated apoptotic resistance is in part mediated through the activated TRAF6-NF-κB signaling. (**a**) Knockdown of TRAF6 attenuates the LPA2 receptor-mediated protection from adriamycin-induced apoptosis. The LPA1/2 DKO MEFs stably expressing FLAG-LPA2 with scrambled shRNA or TRAF6 shRNA were pretreated with 10 μm LPA for 1 h, followed by addition of 2 μm adriamycin (Adr) for 8 h. Apoptosis was determined by caspase-3/7 activity assay (top panel). Data shown are the mean±s.e.m. of three independent experiments (*P<0.001 versus Adr treatment; **P<0.01 versus LPA2-shScr MEFs treated with Adr and LPA; Student’s *t-test*). Another set of cells was subjected to cell viability assay (bottom panel). After treatment, cells were incubated with 0.5 μm calcein-AM and 0.1 μg ml^−1^ propidium iodide at room temperature for 10 min. Images of calcein-positive green fluorescent live cells and propidium iodide-positive dead cells were acquired under fluorescence microscope. Totally 500–1000 cells per sample were counted to determine the percentage of dead cells. Data shown are the mean±s.e.m. of three independent experiments (*P<0.001, **P<0.01 versus LPA2-shScr MEFs treated with Adr; ***P<0.01 versus LPA2-shScr MEFs treated with Adr and LPA; Student’s *t-test*). (**b**) Depletion of TRIP6 eliminates LPA-mediated protection from cisplatin-induced apoptosis in ovarian cancer cells. SKOV-3 stable cell lines expressing Cas9 (sgControl) or Cas9/TRIP6 sgRNA (sgTRIP6-1, sgTRIP6-2) were pretreated with 10 μm LPA in 0.1% fatty acid-free BSA-containing medium for 1 h, followed by addition of 50 μm cisplatin for 20 h. Apoptosis was determined by caspase-3/7 activity assay (top panel). Data shown are the mean±s.e.m. of three independent experiments (*P<0.01 versus sgControl cells treated with cisplatin; Student’s *t-test*). The percentage of dead cells was determined by cell viability assay using calcein/propidium iodide double fluorescence staining as described above (bottom panel). Data shown are the mean±s.e.m. of three independent experiments (*P<0.05 versus sgControl cells treated with cisplatin; Student’s *t-test*). (**c**) Adding TRIP6 back to the TRIP6-depleted SKOV-3 cells restores the antiapoptotic function of LPA; however, this effect is attenuated by the E52A mutation. SKOV-3 cells stably expressing Cas9/TRIP6 sgRNA (SKOV-3-sgTRIP6-1) were transiently transfected with an empty vector (mock), FLAG-TRIP6 or FLAG-E52A-TRIP6. Cells were pretreated with 10 μm LPA for 1 h, followed by addition of 50 μm cisplatin for 20 h. Apoptosis was determined by caspase-3/7 activity assay (top panel). Data shown are the mean±s.e.m. of three independent experiments (*P<0.01, **P<0.05 versus cisplatin-treated cells; ***P<0.05 versus FLAG-TRIP6 cells treated with cisplatin and LPA; Student’s *t-test*). The percentage of dead cells was determined by cell viability assay using calcein/propidium iodide double fluorescence staining as described above (bottom panel). Data shown are the mean±s.e.m. of three independent experiments (*P<0.05 versus FLAG-TRIP6 cells treated with cisplatin; **P<0.05 versus FLAG-TRIP6 cells treated with cisplatin and LPA; Student’s *t-test*). The right bottom panels are two representative immunoblots showing the expression of FLAG-TRIP6, FLAG-E52A-TRIP6 or GAPDH in the whole-cell lysates. (**d**) The effect of TRIP6 knockdown on the inhibition of LPA-mediated antiapoptotic function can be rescued by transiently overexpressing TRIP6; however, this effect is attenuated by the E52A mutation. SKOV-3 cells stably expressing TRIP6 shRNA were transiently transfected with an empty vector (mock), FLAG-TRIP6 or FLAG-E52A-TRIP6. Cells were treated with 10 μm LPA, followed by addition of 50 μm cisplatin for 20 h. Apoptosis was determined by caspase-3/7 activity assay. Data shown are the mean±s.e.m. of three independent experiments (*P<0.01, **P<0.05 versus cisplatin-treated cells; ***P<0.05 versus FLAG-TRIP6 cells treated with cisplatin and LPA; Student’s *t-test*). The bottom two panels are representative immunoblots showing the expression of endogenous TRIP6, FLAG-TRIP6, FLAG-E52A, or GAPDH in the whole-cell lysates.

We next examined how TRIP6 cooperates with TRAF6 to regulate the LPA2 receptor-mediated apoptotic resistance. Thus, LPA1/2 DKO MEFs stably expressing the LPA2 receptor were treated with adriamycin for 8 h in the absence or presence of LPA. The caspase-3/7 activity and cell viability assays showed that LPA greatly protected cells from adriamycin-induced caspase-3/7 activation and cell death; however, this effect was significantly attenuated by shRNA-mediated knockdown of TRAF6 (Figure 6a). Likewise, the effect of LPA on protecting cells from cisplatin-induced caspase-3/7 activation and cell death was abolished by Cas9/sgRNA-directed disruption of TRIP6 gene in two SKOV-3 stable cell lines (Figure 6b). Nonetheless, transiently adding TRIP6 back to the TRIP6-depleted SKOV-3-sgTRIP6-1 cells was able to restore the antiapoptotic effect of LPA; however, this effect was attenuated by the E52A mutation (Figure 6c). Likewise, the effect of TRIP6 knockdown on attenuating LPA-mediated protection from cisplatin-induced caspase-3/7 activation could be rescued by transiently overexpressing TRIP6; however, this effect was reduced by the E52A mutation (Figure 6d). These results confirm that TRIP6 plays a critical role in LPA-mediated apoptotic resistance, which is in part mediated through the activated TRAF6-NF-κB signaling.

### TRAF6 regulates the functions of TRIP6 in NF-κB p65 binding, c-Src-dependent phosphorylation and LPA2 receptor-mediated cell migration

**Figure 7:**
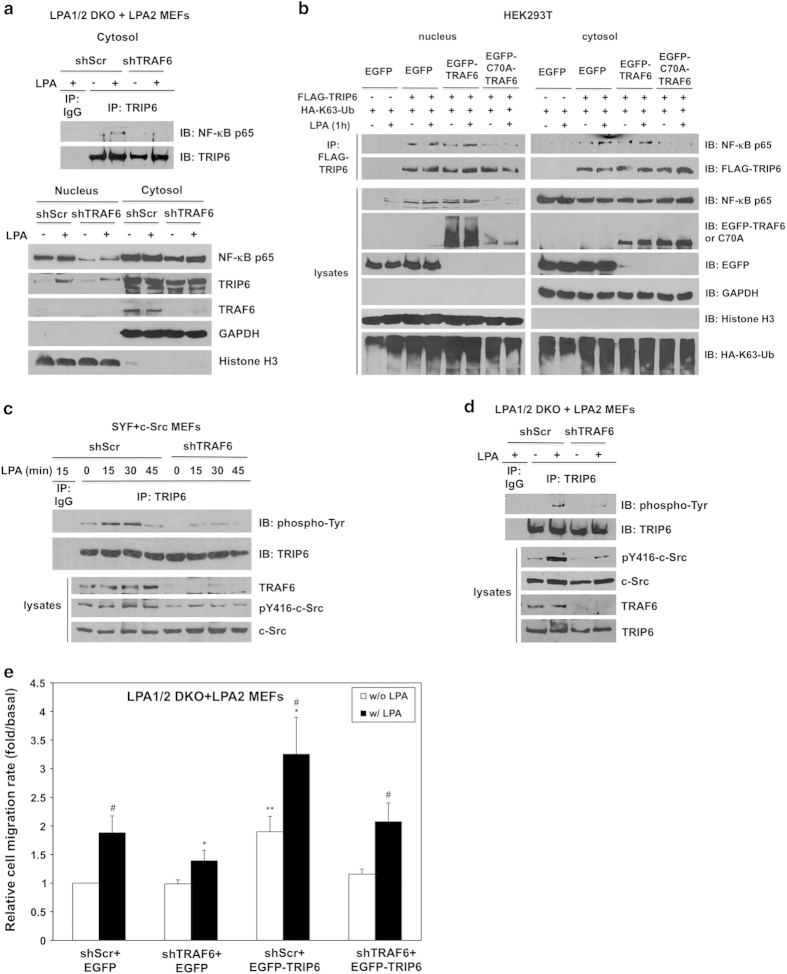
TRAF6 regulates the functions of TRIP6 in NF-κB p65 binding, c-Src-dependent tyrosine phosphorylation of TRIP6 and LPA2 receptor-mediated cell migration. (**a**) Knockdown of TRAF6 eliminates LPA-induced association of TRIP6 with NF-κB p65 in the cytosol, and reduces the levels of nuclear NF-κB p65. LPA1/2 DKO MEFs stably expressing LPA2 receptor and either scrambled shRNA or TRAF6 shRNA were starved for 6 h, followed by LPA stimulation for 1 h. Cells were subjected to subcellular fractionation to separate nuclei from the cytosol. Immunoblotting was performed to detect the expression of NF-κB p65, TRIP6 and TRAF6 in each fraction (bottom panel). Histone H3 and GAPDH serve as nuclear and cytosolic markers, respectively. The cytosolic TRIP6 was further immunoprecipitated with anti-TRIP6 mouse monoclonal antibody or control mouse IgG, followed by immunoblotting using anti-NF-κB p65 rabbit antibody to detect the association of TRIP6 with NF-κB p65 in the cytosol (top panel). (**b**) Overexpression of ligase-defective C70A-TRAF6 eliminates the association of TRIP6 with NF-κB p65. HEK293T cells transiently expressing FLAG-TRIP6, HA-K63-ubiquitin and either EGFP, EGFP-TRAF6 or EGFP-C70A-TRAF6 were starved for 6 h, followed by LPA stimulation for 1 h. Subcellular fractionation was performed in hypotonic buffer to separate nuclei (pellet) from the cytosol (supernatant). FLAG-TRIP6 in each fraction was immunoprecipitated with anti-FLAG M2 mouse monoclonal antibody-conjugated agarose beads, followed by immunoblotting to detect co-immunoprecipitated endogenous NF-κB p65. The immunoblot was reprobed with anti-FLAG antibody to detect precipitated FLAG-TRIP6. The expression of HA-ubiquitin or EGFP fusion proteins in each fraction was detected by immunoblotting using anti-HA or anti-GFP antibody. GAPDH and Histone H3 serve as cytosolic and nuclear markers, respectively. (**c** and **d**) Knockdown of TRAF6 attenuates c-Src kinase activity and reduces LPA-stimulated tyrosine phosphorylation of TRIP6. SYF+c-Src MEFs (**c**) or LPA1/2 DKO+LPA2 MEFs (**d**) were infected with lentivirus harboring scrambled shRNA or mouse TRAF6-specific shRNA. Cells were starved for 8 h, followed by LPA stimulation for the indicated times. Endogenous TRIP6 was immunoprecipitated with anti-TRIP6 mouse monoclonal antibody, followed by immunoblotting using HRP-conjugated anti-phospho-tyrosine antibody and anti-TRIP6 rabbit antibody, respectively. The bottom panels show the expression of pY416-c-Src, total c-Src, TRAF6 or TRIP6 in the whole-cell lysates. Data shown in (**a**–**d**) are representative of two to four independent experiments. (**e**) Knockdown of TRAF6 reduces LPA-induced cell migration and attenuates the function of TRIP6 in promoting the LPA2 receptor-mediated cell migration. The LPA1/2 DKO MEFs stably expressing FLAG-LPA2 receptor and either scramble shRNA or TRAF6 shRNA were transduced with lentivirus harboring EGFP or EGFP-TRIP6. LPA was added to the lower chamber of transwells, and cells were allowed to migrate for 6 h. Cells that migrated to the bottom filters of the transwells were fixed and stained with crystal violet. The relative migration rate was defined as the fold-increase of migrated cells compared to untreated shScr+EGFP cells. Data shown are the mean±s.e.m. of three independent experiments (*P<0.05 versus treated shScr+EGFP cells; **P<0.05 versus untreated shScr+EGFP cells; ^#^*P*<0.05 versus untreated cells; Student’s *t-test*).

In addition to binding to TRAF6, TRIP6 can also interact with NF-κB p65 in the cytosol and promote its nuclear translocation following prolonged LPA stimulation [21], raising the possibility whether these events are dependent on the TRAF6 activity. To test this hypothesis, we first examined the effect of TRAF6 knockdown on the association of TRIP6 with NF-κB p65 in the cytosol in LPA1/2 DKO MEFs stably expressing FLAG-LPA2 receptor. Using subcellular fractionation to enrich the cytosolic fraction, we found that knockdown of TRAF6 eliminated LPA-induced association of TRIP6 with NF-κB p65 in the cytosol (Figure 7a, top panel), indicating that this binding requires TRAF6. It was apparent that knockdown of TRAF6 greatly reduced the levels of nuclear NF-κB p65 in the absence or presence of LPA, bud did not affect LPA-promoted nuclear translocation of TRIP6 (Figure 7a, bottom panel).

To further address if the E3 ligase activity of TRAF6 is required for the binding of TRIP6 to NF-κB p65, TRIP6 and HA-K63-ubiquitin were coexpressed with either wild-type TRAF6 or ligase-dead C70A-TRAF6 in HEK293T cells. We found that overexpression of TRIP6 increased the levels of nuclear NF-κB p65 in HEK293T cells without or with LPA treatment for 1 h (Figure 7b). Although this effect was not further enhanced by wild-type TRAF6, it was blocked by C70A-TRAF6 mutant. Under this condition, the association of TRIP6 with NF-κB p65 was not affected by wild-type TRAF6, but was eliminated by C70A-TRAF6 in the nucleus as well as in the cytosol, indicating that the E3 ligase activity of TRAF6 is required for this complex assembly. Since TRIP6 not only regulates TRAF6 in the cytosol, but can also serve as a coactivator of NF-κB p65 and c-fos in the nucleus [25, 33], these sequential events indeed provide dual mechanisms for the amplification of NF-κB and AP-1 transcriptional activity.

**Figure 8:**
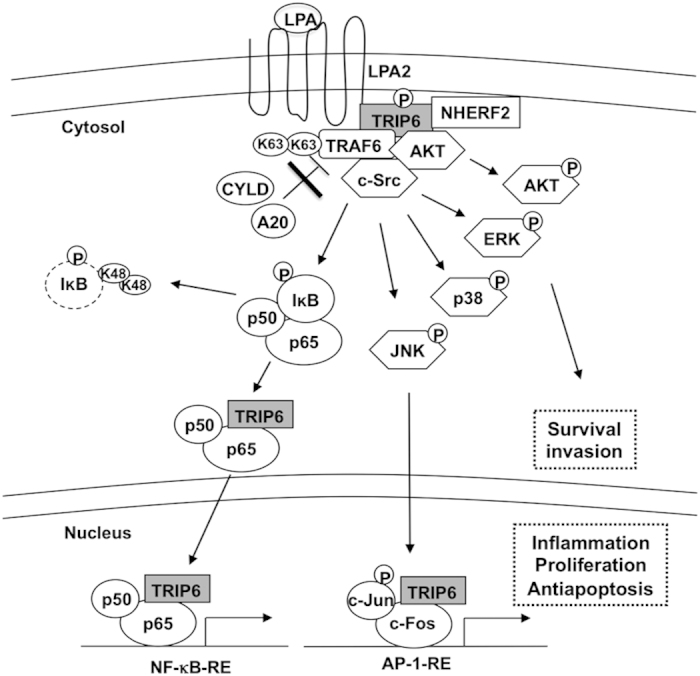
A model for the cooperative regulation between TRIP6 and TRAF6 in the LPA2 receptor signaling. Upon LPA stimulation, TRIP6 is targeted to the plasma membrane, where it forms a complex with the LPA2 receptor and NHERF2, and serves as a scaffold to recruit TRAF6, c-Src and AKT. Together, they coordinate to activate ERK, JNK, p38, AKT and induce c-Src-dependent tyrosine phosphorylation of TRIP6. Through direct binding to TRAF6, TRIP6 antagonizes the recruitment of A20 and CYLD to TRAF6, thus sustaining the E3 ligase activity of TRAF6 upon LPA stimulation. This promotes the IKK-dependent phosphorylation and degradation of IκB, and allows TRIP6 to bind NF-κB p65. Once translocated to the nucleus, TRIP6 can further serve as a coactivator of NF-κB p65 and AP-1 to activate their target genes involved in apoptotic resistance, chronic inflammation, proliferation and invasion.

Previously we have shown that c-Src-dependent phosphorylation of TRIP6 at Tyr-55 is required for its coupling to Crk and regulation of LPA-induced cell migration [34]. As TRAF6 has been shown to interact with and promote the kinase activity of Src family kinases [35], it is possible that TRAF6 may promote the LPA2 receptor-mediated cell migration through regulation of tyrosine phosphorylation of TRIP6. To address this possibility, we first examined the effect of TRAF6 knockdown on LPA-induced c-Src activation in Src/Yes/Fyn-null SYF MEFs stably overexpressing c-Src (designated SYF+c-Src). Indeed, knockdown of TRAF6 in SYF+c-Src MEFs attenuated c-Src activity, as indicated by the reduced Y416 phosphorylation, as well as LPA-induced tyrosine phosphorylation of TRIP6 (Figure 7c). Consistently, knockdown of TRAF6 also attenuated LPA-promoted c-Src activation and tyrosine phosphorylation of TRIP6 (Figure 7d), and significantly decreased LPA-induced cell migration in the LPA1/2 DKO MEFs stably expressing FLAG-LPA2 receptor (Figure 7e). Although overexpression of EGFP-TRIP6 increased the basal motility and LPA-induced cell migration, this effect was attenuated by TRAF6 knockdown (Figure 7e), suggesting that the function of TRIP6 in promoting LPA-induced cell migration is partly dependent on TRAF6. Taken together, these results demonstrate a reciprocal positive regulation between TRIP6 and TRAF6 in the LPA2 receptor-mediated NF-κB and AP-1 signaling, which may ultimately contribute to apoptotic resistance, chronic inflammation, cell proliferation and invasion (Figure 8).

## Discussion

Among the different LPA receptors, the LPA2 receptor has been shown to play a critical role in apoptotic resistance, inflammation and tumor progression in several types of cancer that overexpress the LPA2 receptor, such as ovarian cancer, breast cancer and colon cancer [17, 36, 37]. Our earlier data show that the LPA2 receptor forms a ternary complex with TRIP6 and NHERF2 to mediate activation of ERK and AKT, rendering cells resistant to chemotherapeutic agent-induced apoptosis [18]. In this report, we further demonstrate a unique role for TRIP6 in the regulation of LPA-stimulated activation of NF-κB and JNK-AP-1 signaling through the recruitment of TRAF6 to the LPA2 receptor. Once activated, TRAF6 can further promote the function of TRIP6 in the binding to and activation of NF-κB. These findings point to a critical role for TRIP6 in the regulation of LPA2 receptor-mediated antiapoptotic signaling through different mechanisms. Previously, it has been shown that the recruitment of TRAF6 by the signalosome containing PKC, CARMA3/MALT1/BCL10 complex, and β-arrestin2 serve as a general mechanism for G protein-coupled receptor-mediated NF-κB activation [2, 38]. Since TRAF6 can also be recruited to the LPA2 receptor specifically by TRIP6, this would provide a unique and additional mechanism to further augment the LPA2 receptor-mediated proinflammatory signaling and apoptotic resistance.

Our data show that TRIP6 binds to the carboxyl-terminal domain and RING domain of TRAF6 through its TRAF6-binding motif and LIM domains 1 to 2, respectively. Although LIM domain by itself does not possess the E3 ligase activity, it has been shown that the LIM2 domain of Hic5 binds to the RING domain of Cbl-c, and this binding promotes the E3 ligase activity of Cbl-c [39]. Here we provide another example that through the association with TRAF6, TRIP6 physically interferes with the recruitment of A20 to TRAF6, thus sustaining the E3 ligase activity of TRAF6 and enhancing LPA-induced NF-κB activation. TRIP6 also attenuates the association of CYLD with TRAF6 in unstimulated HEK293T cells, although the effect is much weaker.

Both A20 and CYLD have been shown to act as tumor suppressors in different cell types. The genetic studies show that deletion or inactivating mutation of A20 is associated with constitutive NF-κB activity and this contributes to hematological malignancies, suggesting a tumor suppressor role for A20 in these cancer types [40]. Since TRIP6 is expressed at very low levels in lymphocytes [21], it may not have significant contribution to the constitutive NF-κB activity during lymphomagenesis. On the other hand, in ovarian cancer cells and glioblastoma cells that show persistent NF-κB activity and high levels of TRIP6, both A20 and CYLD bind to TRAF6 very weakly. Nonetheless, depletion of TRIP6 can significantly enhance the association of A20 or CYLD with TRAF6 in ovarian cancer cells and promote the binding of A20 to TRAF6 in glioblastoma cells, suggesting that targeting TRIP6 may prove to be an effective strategy to restore the function of A20 and CYLD in restricting the NF-κB activity in these cancer cells.

It appears that TRAF6 can reciprocally regulate the function of TRIP6 in NF-κB signaling and cell motility. Our data show that the E3 ligase activity of TRAF6 is required for the association of TRIP6 with NF-κB p65. It is likely that TRAF6-catalyzed K63-linked ubiquitination modification can either regulate the association of TRIP6 with NF-κB p65 directly, or promote the phosphorylation and degradation of IκBα, thus releasing NF-κB p65 to bind TRIP6 and further activating the NF-κB signals. These data suggest a reciprocal positive regulatory loop between TRIP6 and TRAF6 in the NF-κB signaling. Moreover, through the complex formation with both c-Src and TRIP6, TRAF6 can also regulate c-Src activity and c-Src-dependent Tyr-55 phosphorylation of TRIP6, thereby promoting its function in the LPA2 receptor-mediated cell migration.

In summary, our present and prior data favor the model (Figure 8) that upon LPA stimulation, TRIP6 is targeted to the plasma membrane, where it forms a ternary complex with the LPA2 receptor and NHERF2. TRIP6 can further serve as a platform to recruit TRAF6, c-Src and AKT, which in turn activate the JNK, p38, ERK and AKT signaling pathways and promote c-Src-dependent tyrosine phosphorylation of TRIP6. Through direct interaction with TRAF6, TRIP6 antagonizes the recruitment of deubiquitinases A20 and CYLD to TRAF6, thus sustaining the E3 ligase activity of TRAF6. These signaling events lead to the IKK-dependent phosphorylation and degradation of IκB, and allow TRIP6 to bind NF-κB p65. Once translocated to the nucleus, TRIP6 can further serve as a coactivator of both NF-κB p65 and AP-1 to promote expression of their target genes involved in inflammation, antiapoptosis, cell proliferation and invasion, which may ultimately contribute to chronic inflammation and tumorigenesis.

## Materials and Methods

### Plasmid construction and transfection

The cDNA sequences of TRAF6 or its truncation mutant containing either the RING domain (residues 1–149), zinc-finger domains (residues 119–238) or carboxyl-terminal domain (residues 239–530) were amplified by polymerase chain reaction using pCMV5-FLAG-TRAF6 (Addgene, Cambridge, MA, USA) [41] as the template, and were inserted in-frame into pEGFP-C1 (Clontech, Mountain View, CA, USA) or pGEX-6P3 (Amersham Biosciences, Piscataway, NJ, USA). The FLAG-E52A-TRIP6 expression vector was constructed by QuikChange site-directed mutagenesis (Agilent, Santa Clara, CA, USA) using pCMV-FLAG-TRIP6 as the template. The cDNA sequences of E52A-TRIP6 were further removed and inserted in-frame into pEGFP-C1. The cDNA sequences of A20 were removed from pEGFP-A20 (Addgene) [42] and inserted in-frame into pCMV-Tag2A (Agilent) to construct pCMV-FLAG-A20. All of the cDNA constructs have been verified by DNA sequencing. The expression vectors of TRIP6 truncation mutants were constructed as described before [23].

The SKOV-3 cell lines stably expressing scrambled shRNA or human TRIP6 shRNA, and the spontaneously immortalized *LPA1*^*−/−*^*, LPA2*^*−/−*^ mouse embryonic fibroblasts (LPA1/2 DKO MEFs) were established as described previously [18, 21]. The LPA1/2 DKO MEFs stably expressing FLAG-LPA2 receptor were further transduced with lentivirus harboring scrambled shRNA, mouse TRIP6 shRNA [18] or mouse TRAF6 shRNA (Sigma Aldrich, St Louis, MO, USA) to establish LPA2-shScr, LPA2-shTRIP6 or LPA2-shTRAF6 MEFs. The Src/Yes/Fyn-null SYF MEFs stably expressing c-Src (designated SYF+c-Src) (American Tissue Culture Collection, Manassas, VA, USA) were transduced with lentivirus harboring scrambled shRNA or mouse TRAF6 shRNA (Sigma Aldrich).

To disrupt TRIP6 gene, a CRISPR/Cas9/single guide RNA system was designed to target exon 2 of TRIP6 using the optimized design software at http://crispr.mit.edu. The 20-nt guide sequence (T1: 5′-CACGCCGGCCCCCGATCCTC-3′ or T2: 5′-GCGTGTGCTGGAGTACTCCA-3′) was cloned into pLentiCRISPRv2 containing a Cas9 expression cassette (Addgene) [43, 44]. The lentivirus harboring Cas9 alone or Cas9/TRIP6 sgRNA was transduced into SKOV-3 or U373-MG cells. After puromycin (3 μg ml^−1^) selection, control stable cells (sgControl) were pooled, and single clones stably expressing Cas9/TRIP6 sgRNA (sgTRIP6) were isolated. The efficiency of TRIP6 knockout was determined by immunoblotting using anti-TRIP6 rabbit antibody (Bethyl Laboratories, Montgomery, TX, USA). Two SKOV-3 stable cell lines (sgTRIP6-1, sgTRIP6-2) harboring TRIP6 sgRNA (T1) and two U373-MG stable cell lines (sgTRIP6-3, sgTRIP6-4) harboring TRIP6 sgRNA (T2) were propagated for further studies.

### Immunoblotting, co-immunoprecipitation, and *in vitro* GST pulldown

Experimental procedures of immunoblotting, co-immunoprecipitation, and purification of recombinant glutathione-*S*-transferase (GST)-tagged or non-tagged proteins were performed as described previously [19, 34]. Immunoblotting was performed using antibody specific to human TRIP6, A20 (Bethyl Laboratories), pT183/Y185-JNK, pT202/Y204-ERK, pS32/36-IκBα, pS176/180-IKKα/β, pY416-c-Src, CYLD, IκBα, IKKβ, Histone H3 (Cell Signaling, Danvers, MA, USA), pT180/Y182-p38 (Promega, Madison, WI, USA), ERK, JNK, mouse TRIP6 (BD Biosciences, San Jose, CA, USA), HA (Cayman, Ann Arbor, MI, USA), FLAG (Sigma-Aldrich), GFP, TRAF6, p38, c-Src, GAPDH, GST, ubiquitin, or phospho-tyrosine (Santa Cruz Biotechnology, Dallas, TX, USA).

### Cellular and *in vitro* ubiquitination assays

To detect K63-linked polyubiquitination of TRAF6, SKOV-3 cells harboring scrambled shRNA or TRIP6 shRNA were transiently transfected with pCMV5-FLAG-TRAF6 (Addgene) [41], pRK5-HA-K63-ubiquitin that encodes a ubiquitin mutant containing only a single K63 with all other lysine residues mutated to Arg (Addgene) [45], and either pEGFP, pEGFP-TRIP6 or pEGFP-E52A-TRIP6. After starvation in 0.1% fatty-acid-free bovine serum albumin (BSA)-containing medium for 6 h, cells were stimulated with 10 μm LPA (Avanti, Alabaster, AL, USA) for 1 h and harvested in boiling RIPA buffer to disrupt protein-protein interactions. After sonication and clarification, equal amounts of cell lysates were subjected to immunoprecipitation with anti-FLAG M2 monoclonal antibody-conjugated agarose beads (Sigma Aldrich), followed by immunoblotting with anti-HA antibody (Cayman) to detect ubiquitinated proteins. To detect K63-linked polyubiquitination of endogenous TRAF6, TRIP6-depleted SKOV-3 or HEK293T cells were transfected with pRK5-HA-K63-ubiquitin. Endogenous TRAF6 in the whole-cell lysates was immunoprecipitated with anti-TRAF6 mouse monoclonal antibody (Santa Cruz Biotechnology), followed by immunoblotting with anti-HA antibody (Cayman) to detect K63-linked polyubiquitnated TRAF6.

The *in vitro* ubiquitination assay was performed by incubating purified recombinant TRAF6 without or with TRIP6 in the presence of purified E1, Ubc13/Uev1A complex, ubiquitin (Boston Biochem, Cambridge, MA, USA) and ATP at 30 °C for 1 h. After heat denaturation in RIPA buffer, immunoprecipitation was performed with anti-TRAF6 monoclonal antibody (Santa Cruz Biotechnology), followed by immunoblotting using anti-ubiquitin antibody (Santa Cruz Biotechnology) to detect ubiquitinated TRAF6.

### Subcellular fractionation

To enrich plasma membrane and cytosolic fractions, cells were scraped into a buffer containing 0.25 m sucrose, 10 mm Tris, pH 7.4, 1 mm EDTA. After dounce homogenization, differential centrifugation was performed as described previously [46]. To separate the nuclear and cytosolic fractions, cells were harvested in hypotonic solution and subjected to differential centrifugation as described before [47]. The nuclear (pellet) and cytosolic (supernatant) fractions were verified by immunoblotting using antibody specific to Histone H3 and GAPDH, respectively.

### Apoptosis and cell viability assays

SKOV-3 or MEF cell lines were pretreated with 10 μm LPA (Avanti) for 1 h in 0.1% fatty acid-free BSA-containing medium, followed by incubation with 50 μm cisplatin for 20 h (SKOV-3) or 2 μm adriamycin or 8 h (MEFs). Caspase-3/7 activity was determined by cleavage of the luminogenic substrate containing the DEVD sequence (Promega) and was normalized to protein concentrations. Alternatively, cell viability was determined by calcein/propidium iodide double fluorescence staining of the live/dead cells. After treatment was complete, cells were incubated with 0.5 μm calcein-AM (AnaSpec, Fremont, CA, USA) and 0.1 μg ml^−1^ propidium iodide (Roche, Indianapolis, IN, USA) for 10 min at room temperature. Images of green fluorescent calcein-positive live cells and red fluorescent propidium iodide-positive dead cells were acquired under fluorescence microscope using ×20 long-ranged objectives. Totally 500–1000 cells per sample were counted to determine the percentage of live and dead cells.

### NF-κB and AP-1 activity assays

SKOV-3 or MEF cell lines were transiently transfected with indicated plasmids, the pCH110 β-galactosidase expression vector, and either pNF-κB-Luc (Agilent), pIL-6-Luc [48], pIL-6 mut-Luc with mutation in the NF-κB-binding site [48], or pGL3-3xAP-1-Luc [49] (Addgene). Cells were starved for 5 h (MEFs) or 24 h (SKOV-3), followed by stimulation with 10 μm LPA for 3 h. Luciferase activity was measured following the manufacturer’s instruction (Promega), and was normalized to the β-galactosidase activity.

### Transwell cell migration assay

The LPA1/2 DKO MEFs stably expressing FLAG-LPA2 receptor with either scrambled shRNA or TRAF6 shRNA were transduced with lentivirus harboring EGFP or EGFP-TRIP6. Ten μm LPA (Avanti) was added to the lower chamber of transwells, and cells were allowed to migrate through 8 μm-pore inserts for 6 h. After removal of the non-migratory cells on the top chambers, the fibronectin-coated bottom filters were fixed and migrated cells were stained with crystal violet. A total of 25 fields/sample were counted under the microscope using a ×20 long-range objective. The relative migratory rate was determined as the fold increase compared to unstimulated control cells.
