# Supplementary material for: TRIP6 antagonizes the recruitment of A20 and CYLD to TRAF6 to promote the LPA2 receptor-mediated TRAF6 activation
Source: Cell Discov. 2016 Mar 1;2:15048–. doi: 10.1038/celldisc.2015.48 (PMC4850058; doi:10.1038/celldisc.2015.48)
Supplement: Supplementary Figure S1 [file celldisc201548-s1.pdf]

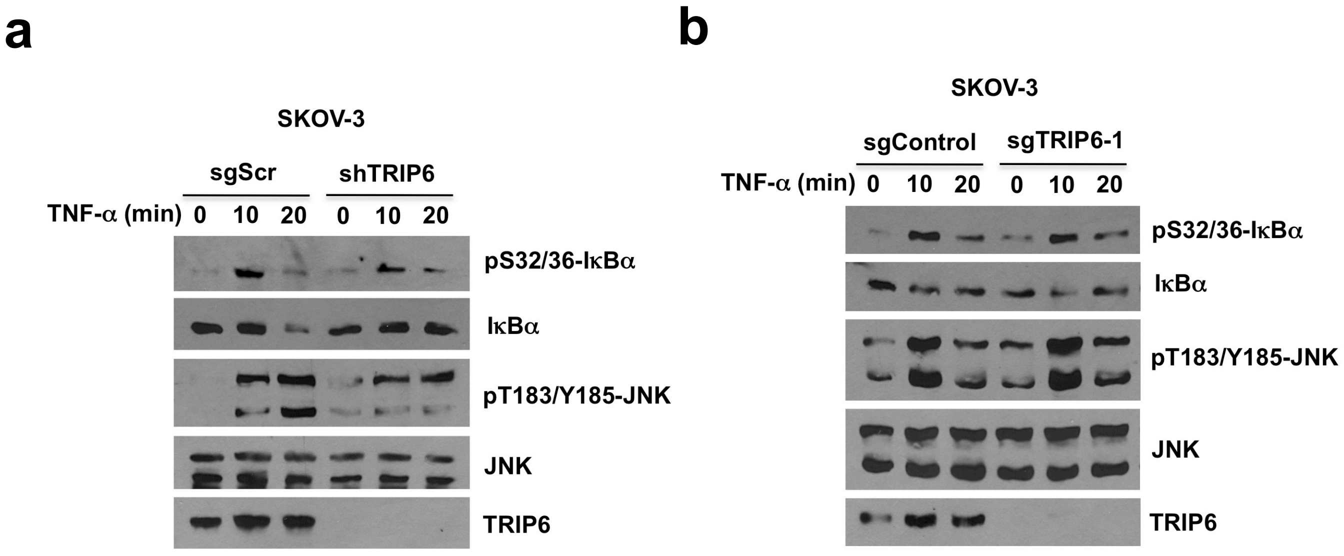

Supplementary Figure S1. Depletion of TRIP6 only mildly or barely affects TNF-α-induced IκBα phosphorylation and JNK activation. SKOV-3 cells stably expressing shRNA (shScr, shTRIP6) (a) or Cas9/sgRNA (sgControl, sgTRIP6-1) (b) were treated with 10 ng/ml TNF-α in 10% fetal bovine serum-containing medium for 10 or 20 min. Immunoblotting was performed to detect the levels of phosphorylated or total IκBα, JNK or TRIP6 in the whole cell lysates.
