# Supplementary material for: TRIP6 antagonizes the recruitment of A20 and CYLD to TRAF6 to promote the LPA2 receptor-mediated TRAF6 activation
Source: Cell Discov. 2016 Mar 1;2:15048–. doi: 10.1038/celldisc.2015.48 (PMC4850058; doi:10.1038/celldisc.2015.48)
Supplement: Supplementary Figure S2 [file celldisc201548-s2.pdf]

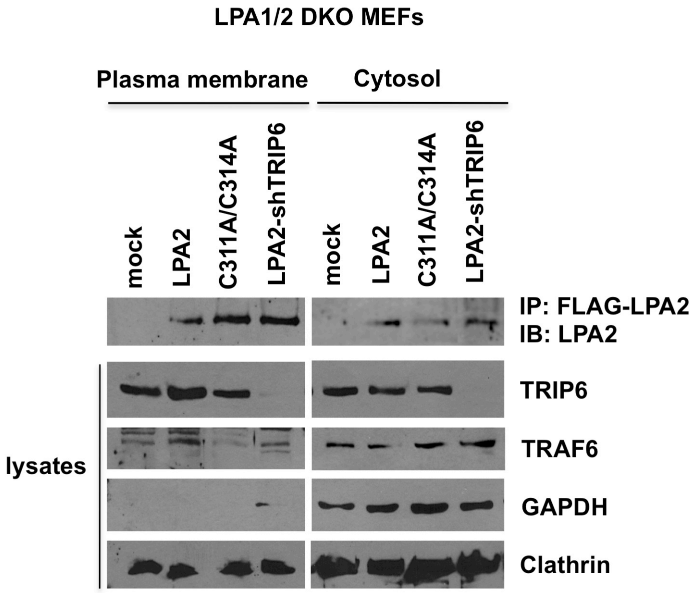

Supplementary Figure S2. Knockdown of TRIP6 or disruption of TRIP6 binding to the LPA2 receptor does not impair the expression of LPA2 receptor on the plasma membrane. LPA1/2 DKO MEFs stably expressing FLAG-LPA2, C311A/C314A mutant FLAG-LPA2 or FLAG-LPA2 with TRIP6 shRNA were harvested in a buffer containing 0.25 M sucrose, 10 mM Tris, pH 7.4, and 1 mM EDTA, pH 8. After dounce homogenization, nuclei were removed and differential centrifugation was performed to separate plasma membrane from the cytosol. FLAG-LPA2 receptor in each fraction was immunoprecipitated with anti-FLAG M2 monoclonal antibody-conjugated agarose beads, followed by immunoblotting using anti-LPA2 rabbit antibody. The bottom four panels show the expression of TRIP6, TRAF6, GAPDH or Clathrin in each fraction.
