# Supplementary material for: TRIP6 antagonizes the recruitment of A20 and CYLD to TRAF6 to promote the LPA2 receptor-mediated TRAF6 activation
Source: Cell Discov. 2016 Mar 1;2:15048–. doi: 10.1038/celldisc.2015.48 (PMC4850058; doi:10.1038/celldisc.2015.48)
Supplement: Supplementary Figure S3 [file celldisc201548-s3.pdf]

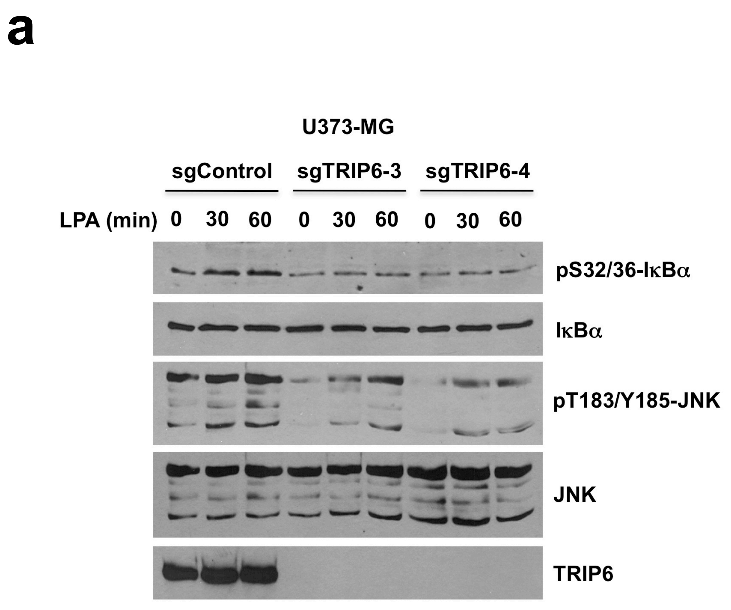

Supplementary Figure S3. Depletion of TRIP6 increases the association of TRAF6 with A20 and attenuates LPA-induced IκBα phosphorylation and JNK activation in U373-MG cells.

(a) Depletion of TRIP6 attenuates LPA-induced IκBα phosphorylation and JNK activation in U373-MG cells. U373-MG stable cell lines (sgControl, sgTRIP6-3, sgTRIP6-4) were starved for 24 h, and then treated with LPA for 30 or 60 min. Immunoblotting was performed to detect the expression of phosphorylated or total IκBα, JNK or TRIP6 in the whole cell lysates.

(b) Depletion of TRIP6 promotes the association of TRAF6 with A20 in U373-MG cells. U373-MG stable cell lines harboring Cas9 alone (sgControl) or Cas9/TRIP6 sgRNA (sgTRIP6-3) were starved overnight, followed by LPA stimulation for 90 min. Endogenous TRAF6 was immunopreipicated with anti-TRAF6 mouse monoclonal antibody or control mouse IgG, followed by immunoblotting using rabbit antibody specific to A20, CYLD or TRAF6. The bottom three panels show the expression of endogenous A20, CYLD or TRIP6 in the whole cell lysates.
